# Different supplements improve insulin resistance, hormonal functions, and oxidative stress on overweight and obese women with polycystic ovary syndrome: a systematic review and meta-analysis

**DOI:** 10.3389/fendo.2024.1464959

**Published:** 2024-12-11

**Authors:** Xiaoyan Ren, Wenjuan Wu, Qiufan Li, Wen Li, Xujuan Wang, Gang Wang

**Affiliations:** Department of Gynecological Oncology, Sichuan Provincial Women’s and Children’s Hospital/The Affiliated Women's and Children's Hospital of Chengdu Medical College, Chengdu, China

**Keywords:** polycystic ovary syndrome, overweight, supplements, insulin resistance, hyperandrogenemian

## Abstract

**Objectives:**

To investigate various supplements that improve insulin resistance, hormonal status, and oxidative stress in overweight or obese women with polycystic ovarian syndrome (PCOS).

**Methods:**

A literature search was conducted on four different databases, which led to the discovery of twenty - five randomized controlled trials (RCTs). These RCTs evaluated the efficacy of various supplements in improving insulin resistance (IR), hormonal status, and oxidative stress among overweight or obese women diagnosed with PCOS. Subsequently, data extraction and analysis were carried out to determine the quality of the study’s methodological design and the potential for bias. Moreover, a meta-analysis was performed using the data from the RCTs.

**Results:**

A total of 25 RCTs were carried out, and 1636 women were enrolled. All participants were overweight or obese. The standardized mean differences (SMD) were as follows: For fasting plasma glucose (FPG), it was -0.34 (95% confidence interval [CI], -0.49 to -0.19, p = 0.123, I^2^ = 30.8%); for insulin, it was -0.67 (95% CI, -0.83 to -0.52, p = 0.208, I^2^ = 24%); for fasting insulin (FI), it was -0.26 (95% CI, -0.52 to -0.00, p = 0.269, I^2^ = 21.9%); for homeostatic model assessment-insulin resistance index (HOMA-IR), it was -0.59 (95% CI, -0.73 to -0.45, p = 0.015, I^2^ = 48.7%); for homoeostatic model assessment beta - cell function (HOMA-B), it was -0.51 (95% CI, -0.75 to -0.27, p = 0.547, I^2^ = 0%); for quantitative insulin sensitivity check index (QUICKI), it was 0.94 (95% CI, 0.76 to -1.12, p = 0.191, I^2^ = 27.5%); for total testosterone, it was -0.61 (95% CI, -1.14 to -0.09, p = 0.00, I^2^ = 78.5%); for testosterone, it was -0.38 (95% CI, -0.86 to 0.10, p = 0.03, I^2^ = 71.5%); for follicle - stimulating hormone (FSH), it was 0.16 (95% CI, -0.08 to 0.40, p = 0.470, I^2^ = 0%); for luteinizing hormone (LH), it was -0.56 (95% CI, -1.32 to 0.20, p = 0.000, I^2^ = 91.1%); for sex hormone - binding globulin (SHBG), it was 0.35 (95% CI, 0.02 to 0.69, p = 0.000, I^2^ = 78%); for dehydroepiandrosterone (DHEAS), it was -0.27 (95% CI, -0.76 to 0.21, p = 0.001, I^2^ = 78.7%); for plasma total antioxidant capacity (TAC), it was 0.87 (95% CI, 0.45 to 1.30, p = 0.004, I^2^ = 71.3%); for plasma malondialdehyde (MDA), it was -0.57 (95% CI, -0.79 to -0.36, p = 0.992, I^2^ = 0.0%).

**Conclusion:**

This study’s findings indicate that, in comparison with a placebo, supplements have a favorable effect on IR, hormonal functions, and oxidative stress in PCOS. Nevertheless, it is crucial to note that the above-drawn conclusions need to be verified by more high-quality studies, given the limitations regarding the number and quality of the included studies.

## 1 Introduction

PCOS, a complex reproductive endocrine and metabolic disorder, is more likely to affect women of childbearing age. Globally, its incidence ranges from 3–10% (1) A survey shows that 28 percent of women with PCOS in Spain are overweight (2), and most overweight patients suffer from insulin resistance (IR). In the long term, it not only affects the operation of the reproductive endocrine system but also increases the probability of developing problems such as metabolic syndrome, glycosuria (3), endometrial cancer, cardiovascular disease, and hypertension (4, 5). These health problems pose a significant risk to women’s overall well - being.

The pathogenesis of PCOS remains unknown. However, variables such as IR (insulin resistance), hyperandrogenemia (HA), genetics, endocrine disorder, obesity, inflammation, and immune system malfunction are considered to contribute to or aggravate PCOS (4, 6). IR leads to reduced responsiveness and sensitivity to insulin stimulation (7), which is a major factor in the development of the disease. In the insulin signaling pathway (8–10), the inflammatory response, oxidative stress, and gut flora are all related to the formation of IR. It has been found that IR is significantly associated with abnormalities in glucose and lipid metabolism, amino acid metabolism (11), HA, ovulation problems, and changes in the physiological function of the endometrium. In overweight or obese women with PCOS, IR is a factor contributing to excessive fat production and worsening obesity. Moreover, HA can exacerbate IR, which may lead to a vicious cycle of more severe symptoms (12).

PCOS is an endocrine and metabolic disorder. Thus, the key to its long-term management is to address the underlying endocrine and metabolic imbalances and implement appropriate treatment strategies. Therapeutic diets and nutritional supplements have been shown to have positive effects on reducing appetite, increasing satiety, promoting weight loss, and reducing fat mass. Currently, a wide variety of supplements are used in the treatment of obese patients with PCOS, such as inulin, CoQ10, thylakoid, carnitine, omega-3, vitamin D, fennel, quercetin, myo-inositol, synbiotic, green cardamom, folate, calcium, probiotic, green tea, and concentrated pomegranate juice (13), etc. A great deal of clinical research has demonstrated the potential of these supplements in treating insulin resistance, hypertension, oxidative stress, inflammation, and metabolic disorders associated with PCOS.

Regarding the technique and data consolidation, there have been no comprehensive and systematic studies comparing supplements with placebo alone. To determine whether supplements, as opposed to placebo alone, can potentially improve IR, hormonal status, and oxidative stress successfully, the main objective of this study was to conduct a comprehensive review and meta-analysis. In this way, overweight individuals with PCOS may have access to a more effective treatment alternative.

## 2 Methods

Throughout the process of conducting this systematic review, the Preferred Reporting of Items for Systematic Reviews and Meta-analyses (PRISMA) (14) checklist was used. This review has been registered with PROSPERO (CRD 42024544518). There is no review mechanism established for this specific review.

### 2.1 Requirements for participation in this study

#### 2.1.1 Categories of individuals involved

This review included PCOS persons who met the diagnostic criteria for PCOS and had a Body Mass Index (BMI) >25 kg/m^2^. The participants were aged between 18 and 45. The 2003 Rotterdam criteria (15) were used for diagnosing PCOS, which involved one of the following signs and symptoms: (a) absent or infrequent menstrual cycles (at least eight cycles in the past year), (b) symptoms of excessive male hormone levels, either detected by laboratory tests or shown as clinical symptoms, and (c) the presence of polycystic ovaries as confirmed by ultrasound, characterized by more than twelve follicles with a diameter between two and nine millimeters and/or an ovarian volume exceeding ten milliliters. The study excluded those who met the following criteria: Participants were excluded if they were under 18 or over 45 years old, had a BMI of 25 kg/m² or less, had used certain medications or interventions within the past three months (such as oral contraceptives, hormone therapy, weight - loss interventions, probiotics, prebiotics, antacids, or antibiotics), had certain medical conditions (such as autoimmune diseases, cancer, cardiovascular disorders, abnormal liver function, kidney diseases, or other endocrine disorders), were allergic to placebo, adhered to specific diet or exercise programs, were current smokers, or were pregnant.

#### 2.1.2 Types of interventions

The treatments included a variety of supplements, such as inulin, CoQ10, thylakoid, carnitine, omega-3, vitamin D, fennel, quercetin, myo-inositol, synbiotic, green cardamom, folate, calcium, probiotics, green tea and concentrated pomegranate juice.

#### 2.1.3 Types of comparators

Comparators encompassed placebo.

#### 2.1.4 Types of outcomes

The primary variables of interest were changes in indices related to IR (FPG, insulin, FI, HOMA-IR, HOMA-B, QUICKI), hormonal profile (total testosterone, testosterone, FSH, LH, SHBG, DHEAS), and oxidative stress (TAC, MDA). The secondary outcomes involved the assessment of anthropometric variables like weight, BMI, Waist-to-Hip Ratio (WHR), and waist circumference (WC). Moreover, the lipid profile markers measured were plasma triglycerides (TG), total cholesterol (TC), and high - density lipoprotein (HDL) cholesterol.

#### 2.1.5 Types of study

This systematic review merely included RCTs.

### 2.2 Search strategy and study selection

Several English-language databases, including PubMed, EMBASE, Cochrane, and Web of Science, were searched for relevant information. Additionally, information was gathered from ClinicalTrials.gov and relevant papers in reference lists. For this search in PubMed, medical subject headings (MeSH) and keywords such as “Polycystic Ovary Syndrome,” “Overweight,” and “Dietary Supplements” were used. The search covered the period from the inception of each database until March 2024. A comprehensive list of search strategies is provided in the **Appendix**. Initially, all retrieved titles and abstracts were imported into the EndNote X9 library, and duplicates were removed. Subsequently, the titles and abstracts of the papers were assessed, and then the full texts of the articles were examined to determine whether they met the inclusion criteria. Finally, the reference lists of the included papers and relevant reviews were scoured for new potentially useful research. The search and screening methods were independently carried out by two researchers, and any discrepancies were resolved through consultation with a third researcher. After that, a fourth researcher extracted key information from the studies included in the analysis. This information comprised the first author, the publication year, the sample size, the age of the participants, the treatments, the outcome measures, and other relevant elements (**Table 1**).

**Table 1:** Demographic and clinical characteristics.

Ref	Study	Year	Country	Sample size	Mean age	Intervention	Outcomes
(16)	Ziaei	2024	Iran	HPI:25 OEI:25 Placebo:25	HPI:29.4 OEI:28.9 Placebo:28.8	HPI:10g/day, 12weeks OEI:10g/day, 12weeks	F1; F2; F3; F4; F5; F6; F7; F8; F9; F10; F11; F20;
(17)	Taghizadeh	2021	Iran	CoQ10:22 Placebo:21	CoQ10:27.64 Placebo:26	CoQ10:200mg/day, 12weeks	F14;
(18)	Tabriz	2020	Iran	Thylakoid:21 Placebo:23	Thylakoid:31.86 Placebo:32.04	Thylakoid:5 g/day of thylakoid-rich spinach extract, 12weeks)	F1; F2; F3; F12;
(20)	Samimi	2016	Iran	Carnitine:30 Placebo:30	Carnitine:25.5 Placebo:24.8	Carnitine:250 mg/d, 12weeks	F1; F2; F3; F4; F6; F7; F8; F9; F10; F13; F14; F15;
(24)	Mohammadi	2012	Iran	Omega-3:30 Placebo:31	Omega-3:27.3 Placebo:27.7	Omega-3:4 g/d ((4×1000-mg capsules, each capsule contained 180 mg EPA and 120 mg DHA), 8weeks	F1; F2; F3; F6; F8; F9; F10; F12; F13;
(22)	Nadjarzadeh	2021	Iran	HSDF:16 HHPF:16 HSDP:16 HHPP:16	HSDF:28.37 HHPF:27.50 HSDP:28.87 HHPP:29.43	HSDF:hypocaloric standardize diet + fennel (2 capsule/day); HHPF:hypocaloric high-protein diet + fennel (2 capsule/day)	F1; F2; F3; F11; F20;
(23)	Nadjarzadeh	2013	Iran	Omega-3:39 Placebo:39	Omega-3:26.92 Placebo:26.91	omega-3:3gr/day, 8weeks	F11; F18;
(26)	Khorshidi	2018	Iran	quercetin:39 Placebo:39	20-40	quercetin:1,000 mg/day, 12weeks	F1; F2; F3; F4; F6; F11; F13; F18; F19;
(36)	Jamilian	2019	Iran	Chromium + carnitine:27 Placebo:27	Chromium + carnitine:29.6 Placebo:27.4	Chromium + carnitine:200 μg/day chromium picolinate +1000 mg/day carnitine, 12weeks	F1; F2; F4; F6; F7; F8; F9; F10; F13; F14;
(27)	Jafari−Sfdvajan	2018	Iran	Vitamin D:30 Placebo:30	Vitamin D:28.43 Placebo:27.83	Vitamin D:50,000 IU/week of vitamin D3, 12weeks	F1; F2; F11; F15; F20;
(28)	Foroozanfard	2015	Iran	Calcium:26 Vitamin D:26 Calcium + vitamin D:26 Placebo:26	18-40	Calcium:1000 mg calcium/day+ vitamin D placebo/week; vitamin D:50000 IU vitamin D /week +calcium placebo/day; Calcium + vitamin D: 1000 mg calcium/day+ 50000 IU vitamin D/week Placebo:calcium placebo/day +vitamin D placebo/week	F14; F16; F17;
(29)	Darvishi	2021	Iran	Synbiotic:34 Placebo:34	Synbiotic:30.4 Placebo:28.6	Synbiotic:1 capsule/day, 8weeks	F1; F2; F3; F6; F8; F9; F10; F12; F13;
(31)	Cheshmeh	2022	Iran	Green cardamom:99 Placebo:95	Green cardamom: 32.99 Placebo:33.81	Green cardamom: 3g/day, 16weeks	F1; F2; F3; F11; F15; F18; F19; F21;
(33)	Asemi	2014	Iran	Folate b:27 Folate c:27 Placebo:27	Folate b:24.3 Folate c:25.1 Placebo:24.7	Folate b:1 mg folate/day Folate c:5 mg folate/day	F1; F2; F4; F6; F7; F8; F9; F10; F13;
(34)	Asemi	2015	Iran	Calcium:26 Vitamin D:26 Calcium + vitamin D:26 Placebo:26	Calcium:25 Vitamin D:25.6 Calcium + vitamin D:24.9 Placebo:24.3	Calcium: 1000mg/day+placebo(vitamin D /week); vitamin D:50000 IU/week+placebo(Calcium/day); Calcium + vitamin D: 1000mg/day+50000 IU/week	F1; F2; F3; F4; F6; F7; F8; F9; F10; F13;
(40)	Nikrad	2023	Iran	Tylakoid:21 Placebo:23	Tylakoid:31.86 Placebo:32.04	Tylakoid:5g/day, 12weeks	F2;
(38)	Rafraf	2012	Iran	Omega-3:30 Placebo:31	Omega-3:27.33 Placebo:27.73	Omega-3: four 1g capsule/day, 8weeks (Each capsule contained 180 mg EPA and 120 mg DHA)	F1; F2; F3; F6; F12; F13;
(19)	Sangouni	2022	Iran	L-carnitine:31 Placebo:31	L-carnitin:30.7 Placebo:30.8	L -carnitine: 1000mg/day, 12weeks	F1; F2; F3; F4; F6; F7; F8; F9; F10; F11; F13;
(21)	Sadeghi	2020	Iran	Omega-3:32 Placebo:30	18-40	Omega-3:two pills/day, 8 weeks (each pill containing 180 mg of Eicos- apentaenoic acid (EPA) + 120 mg of Docosahexaenoic acid (DHA), + 400 IU vitamin E)	F16; F17;
(25)	Łagowska	2022	Poland	DP(diet+probiotic(Lactobacillus rhamnosus)): 19 D(diet): 21	18-45	DP(diet+probiotic(Lactobacillus rhamnosus) ): 12 × 109 CFU/day, 20weeks	F1; F2; F3; F8; F9; F10;
(32)	Chan	2006	China	Green tea (EGCG):20 Placebo:20	25-40	Green tea:540mg EGCG/day	F1; F2; F4; F5; F11; F12; F15; F19; F20; F21;
(30)	Chudzicka-Strugała	2021	Poland	Synbiotic:20 Placebo:19	Synbiotic:30.8 Placebo:29.1	Synbiotic: 4 capsules/day	F1; F2; F3; F4; F5; F8; F9; F10; F11; F12; F15; F19; F20; F21;
(35)	Abedini	2023	Iran	Concentrated pomegranate juice:21 Placebo:21	concentrated pomegranate juice:24.76 Placebo:25.57	concentrated pomegranate juice: dilute 45 ml of concentrated PJ with 180 ml of water/day, 8weeks	F11; F16; F17; F18; F19;
(39)	Marnani	2020	Iran	SDF:11 HPF:15 SDP:15 HPP:15	SDF:28.72 HPF:28.06 SDP:29.6 HPP:29.86	Fennel:60mg/day, 12 weeks	F4; F5; F6;
(37)	Borzoei	2018	Iran	Cinnamon:42 Placebo:42	Cinnamon:29.26 Placebo:30.17	Cinnamon: 1500mg/day, 8weeks	F2; F16; F17;

F1, Weight; F2, BMI; F3, WC; F4, FPG; F5, FI; F6, HOMA-IR; F7, QUICKI; F8, Triglycerides; F9, Total cholesterol; F10, HDL cholesterol; F11, SHBG; F12, WHR; F13, Insulin; F14, HOMA-B; F15, DHEAS; F16, TAC; F17, MDA; F18, Testosterone; F19, LH; F20, Total testosterone; F21, FSH; BMI, Body Mass Index; WC, waist circumference; FI, fasting insulin; HOMA-IR, homeostatic model assessment-insulin resistance index; WHR, Waist-to-Hip Ratio; HOMA-B, homoeostatic model assessment beta-cell function; QUICKI, quantitative insulin sensitivity check index; TC, total cholesterol; HDL-C, high density lipoprotein cholesterol; TAC, plasma total antioxidant capacity; MDA, malondialde-hyde; SHBG, sex hormone binding globulin; DHEAS, Dehydroepiandrosterone.

### 2.3 Assessment of methodological quality

The Cochrane Handbook V.5.1.0 was used to assess the risk of bias in RCTs. In case of any disagreements, two researchers (WL and FQL) carried out independent evaluations. If a consensus was needed, a third researcher (JXW) would participate. The evaluation covered seven aspects, namely: random sequence generation (related to selection bias), allocation concealment (also related to selection bias), blinding of implementers and participants (related to implementation bias), blinding of outcome assessors (related to observation bias), completeness of outcome data (related to follow-up bias), selective reporting of study results (related to reporting bias), and other biases. According to these criteria, each study was comprehensively evaluated. Studies that met all the criteria were classified as having a ‘low risk’ of bias, meaning they were of good quality with low overall bias. Those that only partially met the criteria were categorized as ‘unclear risk’, indicating a moderate chance of bias. Studies that did not meet any of the criteria were classified as ‘high risk’, suggesting poor quality and a high risk of bias.

### 2.4 Data extraction

During the data extraction process, two researchers independently utilized standardized forms. Should any discrepancies arise, a third researcher was tasked with resolving them. The standardized forms included the following information: authors, publication year, country, research design, subjects, diagnostic methods, sample size, interventions for experimental and control groups, duration, and results. Systematic evaluation encompassed intervention data across a range of parameters. The factors taken into account in this study consisted of various IR indicators (such as FPG, insulin, FI, HOMA-IR, HOMA-B, QUICKI, and DHEAS), hormonal status (such as Total Testosterone, Testosterone, FSH, LH, and SHBG), oxidative stress (such as TAC and MDA), anthropometric outcomes (such as weight, BMI, WHR, and WC), and lipid profiles (such as plasma TG, TC, and HDL). Subsequently, these factors were analyzed in the meta-analysis.

### 2.5 Statistical analysis

To conduct statistical analysis, Stata 15 (Stata Corporation, College Station, Texas, USA) was used. Firstly, clinical heterogeneity was evaluated. If present, the meta-analysis was terminated, and the results were reported descriptively through text and comprehensive tables. After eliminating clinical heterogeneity, the chi-square test was used to further assess the statistical heterogeneity of the subjects. Mean differences (MD) or SMD for continuous variables were calculated with 95% confidence intervals (CI) on each side. A significance threshold of p < 0.05 was used to declare statistical significance. Based on the goals of the investigation, an evaluation was made to determine the degree of similarity in clinical outcome indicators between the intervention group and the control group. The I^2^ test was applied to check for statistical heterogeneity. I^2^ values of 0%, 25%, 50%, and 75% represented no heterogeneity, low heterogeneity, medium heterogeneity, and high heterogeneity respectively. If the heterogeneity was less than 50%, the fixed-effects model was used. Additionally, the Egger test and the random-effects model were used to evaluate the publication bias involved.

## 3 Results

### 3.1 Study selection and description of study characteristics

**Figure 1:**
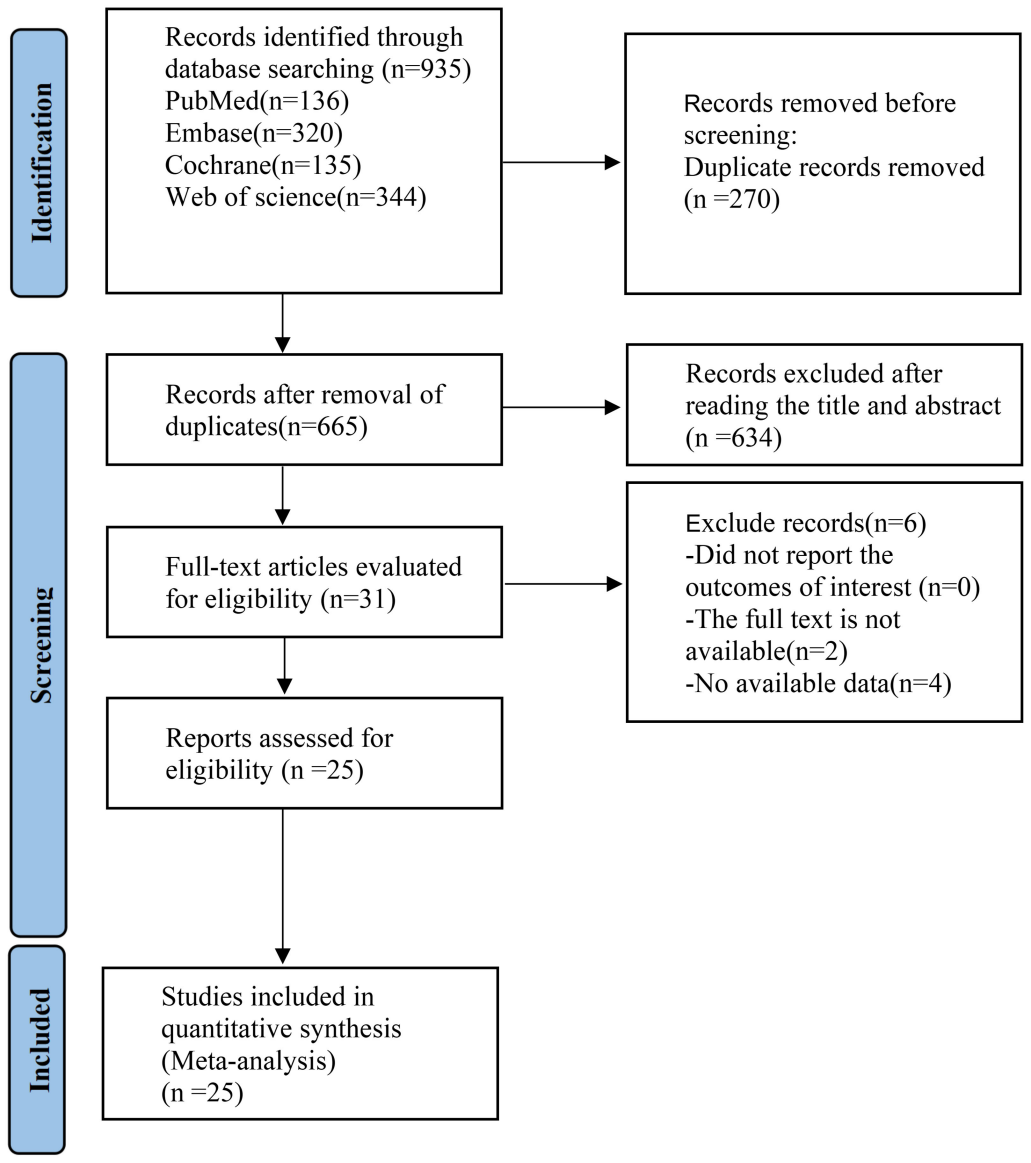
PRISMA flow diagram of the study process.

The PRISMA flow diagram provided an illustration of the selection procedure for the systematic review and meta-analysis. Initially, a total of 935 relevant research studies were evaluated. After multiple rounds of screening, 25 (16–40)studies were eligible for inclusion. The study included a cohort of 925 adults diagnosed with obesity and PCOS. These participants were given supplements as part of 25 RCTs (**Figure 1**). A summary of the included RCTs is shown in **Table 1**.

### 3.2 The quality of the methodology

**Figure 2:**
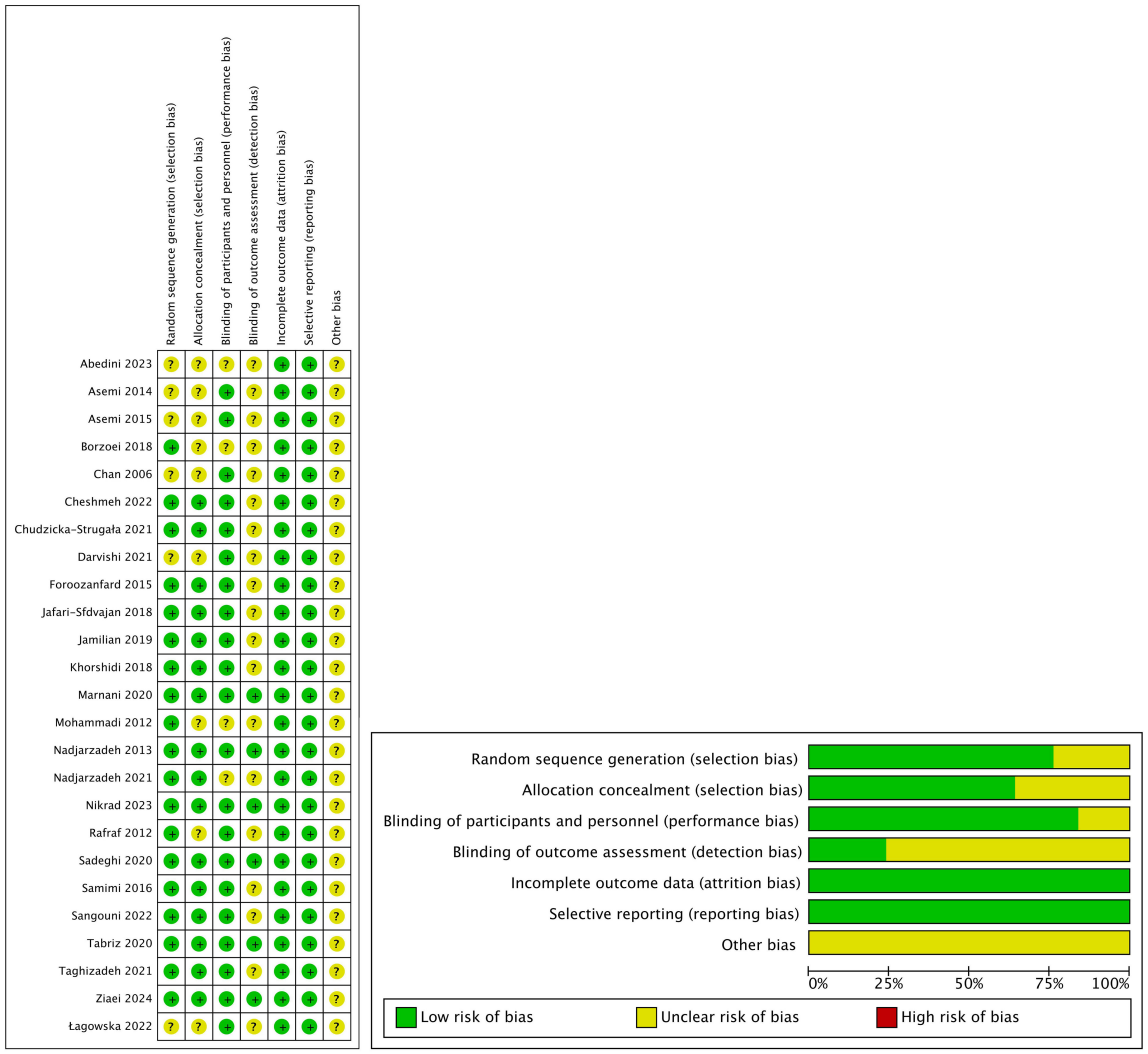
Risk of bias assessed in the included randomized controlled trials. Plus-sign represents ‘low risk’, Question mark represents ‘unclear risk’.

The risk-bias assessment information for each RCT is presented in **Figure 2**. Since other bias is not mentioned in the article, it was regarded as unclear. 6 RCTs (16, 18, 21, 23, 39, 40) were assigned a relatively low risk of bias because information for each item (except other bias) was comprehensively reported. 19 RCTs (17, 19, 20, 22, 24–38) were given an uncertain risk of bias due to insufficient reporting. Among the included studies, 6 RCTs (25, 29, 32–35) lacked sufficient information regarding the random sequence generation method, which might have introduced selection bias. The allocation concealment information of 9 RCTs (24, 25, 29, 32–35, 37, 38) was incomplete, and the design and blinding implementation of 4 RCTs (22, 24, 35, 37) were not clear. Also, the blinding of 19 RCT (17, 19, 20, 22, 24–38) outcome assessors was unclear.

### 3.3 Impacts of interventions

#### 3.3.1 FPG and insulin resistance related index

**Figure 3:**
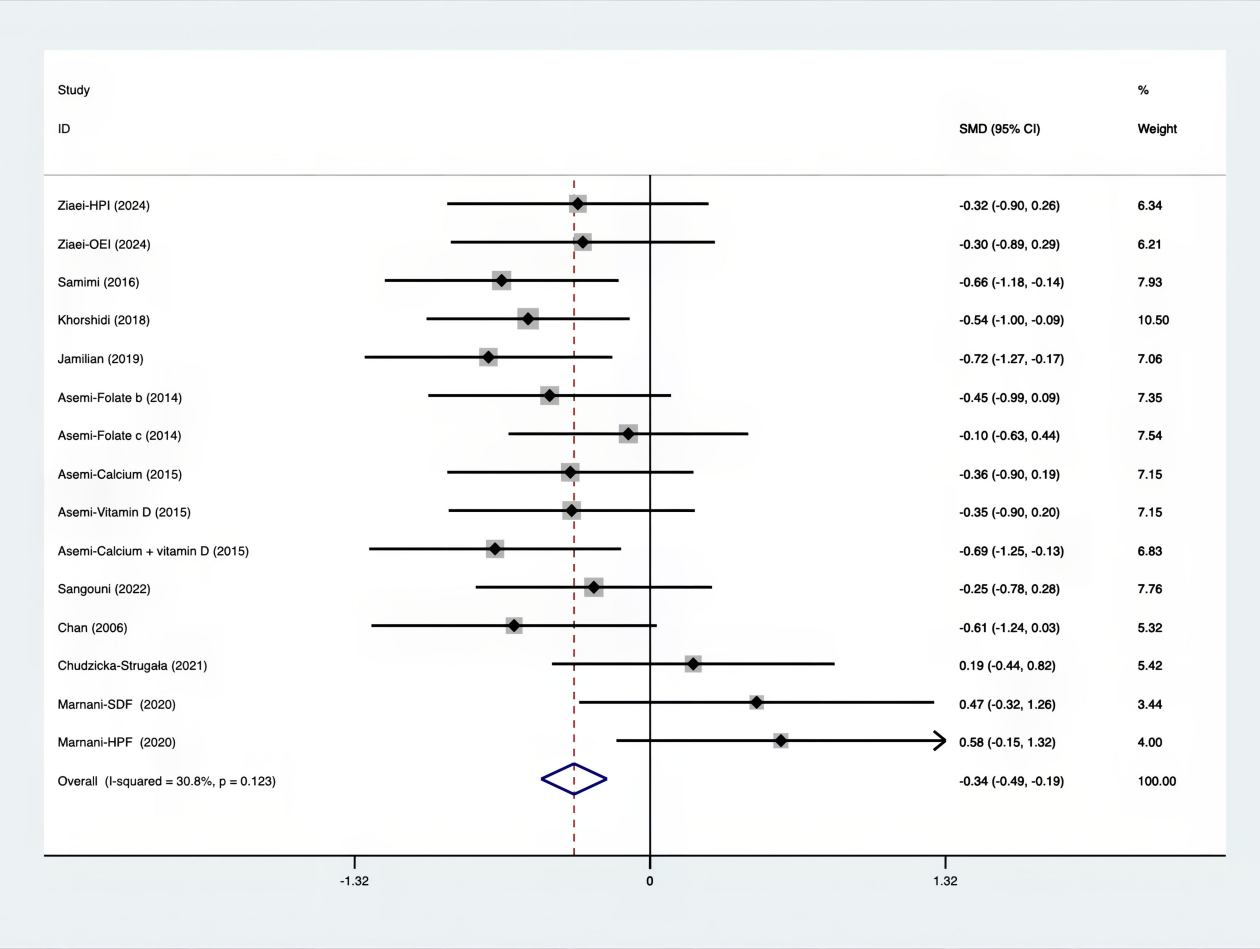
Forest plot of supplements versus placebo on FPG.

**Figure 4:**
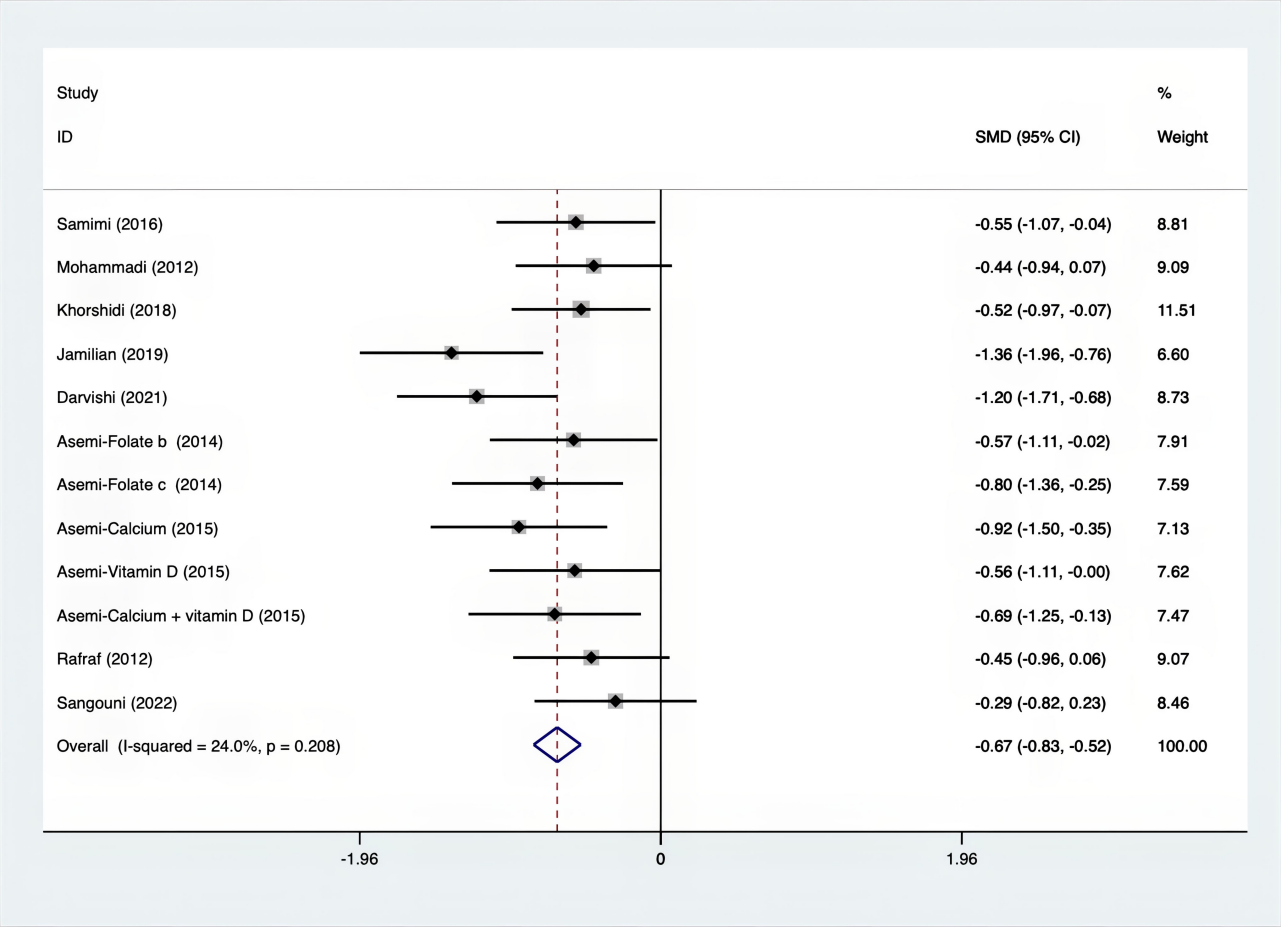
Forest plot of supplements versus placebo on insulin.

**Figure 5:**
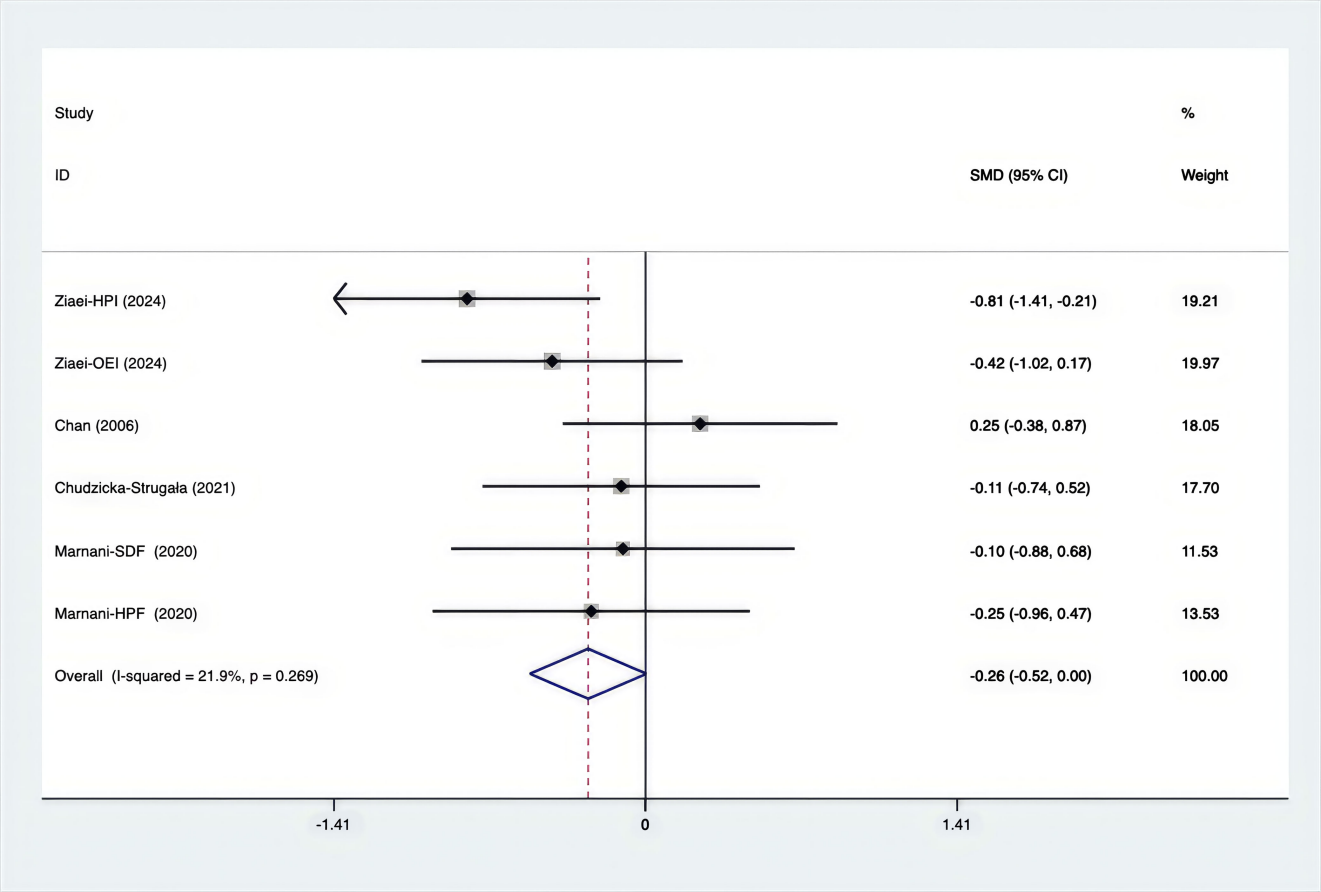
Forest plot of supplements versus placebo on FI.

**Figure 6:**
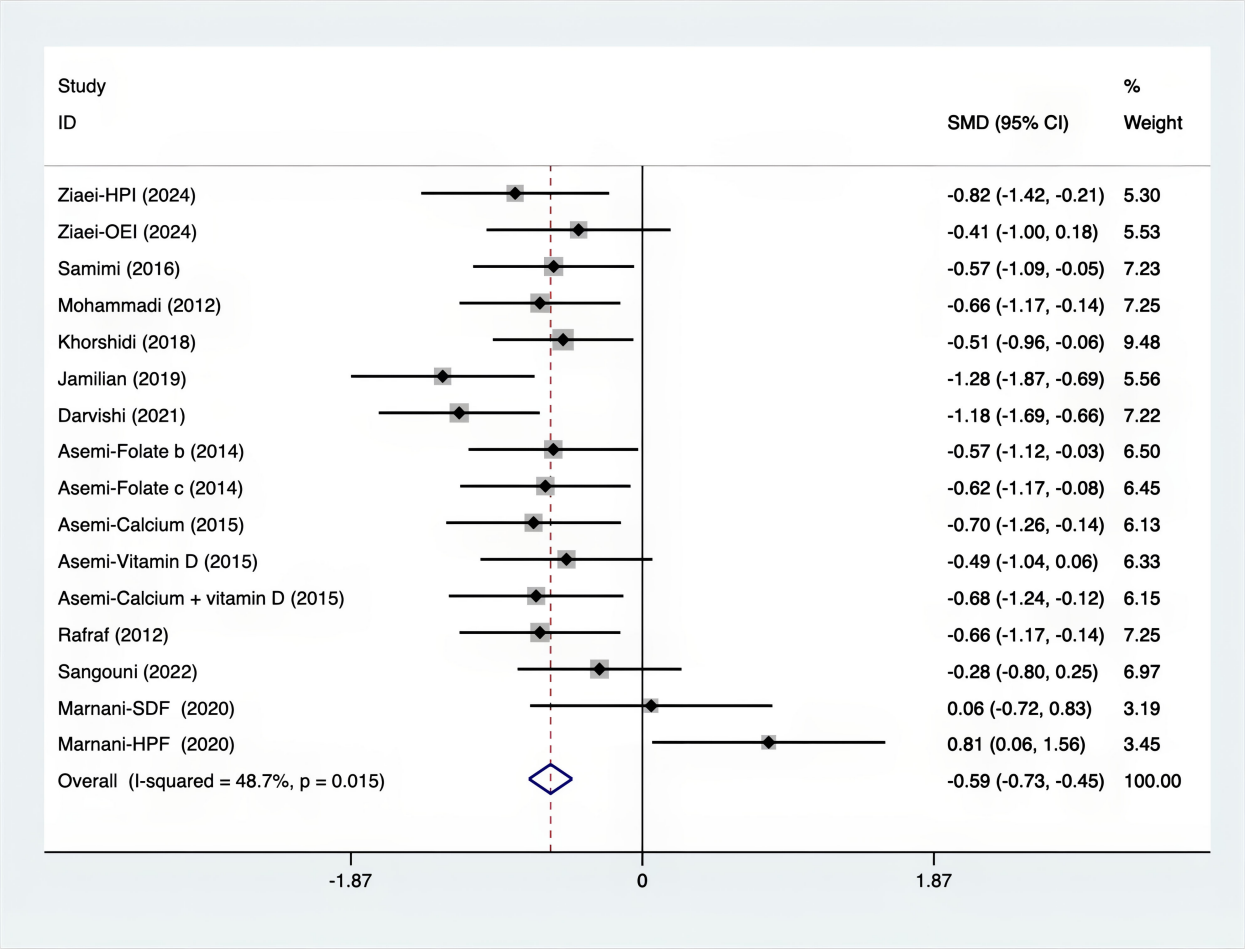
Forest plot of supplements versus placebo on HOMA-IR.

**Figure 7:**
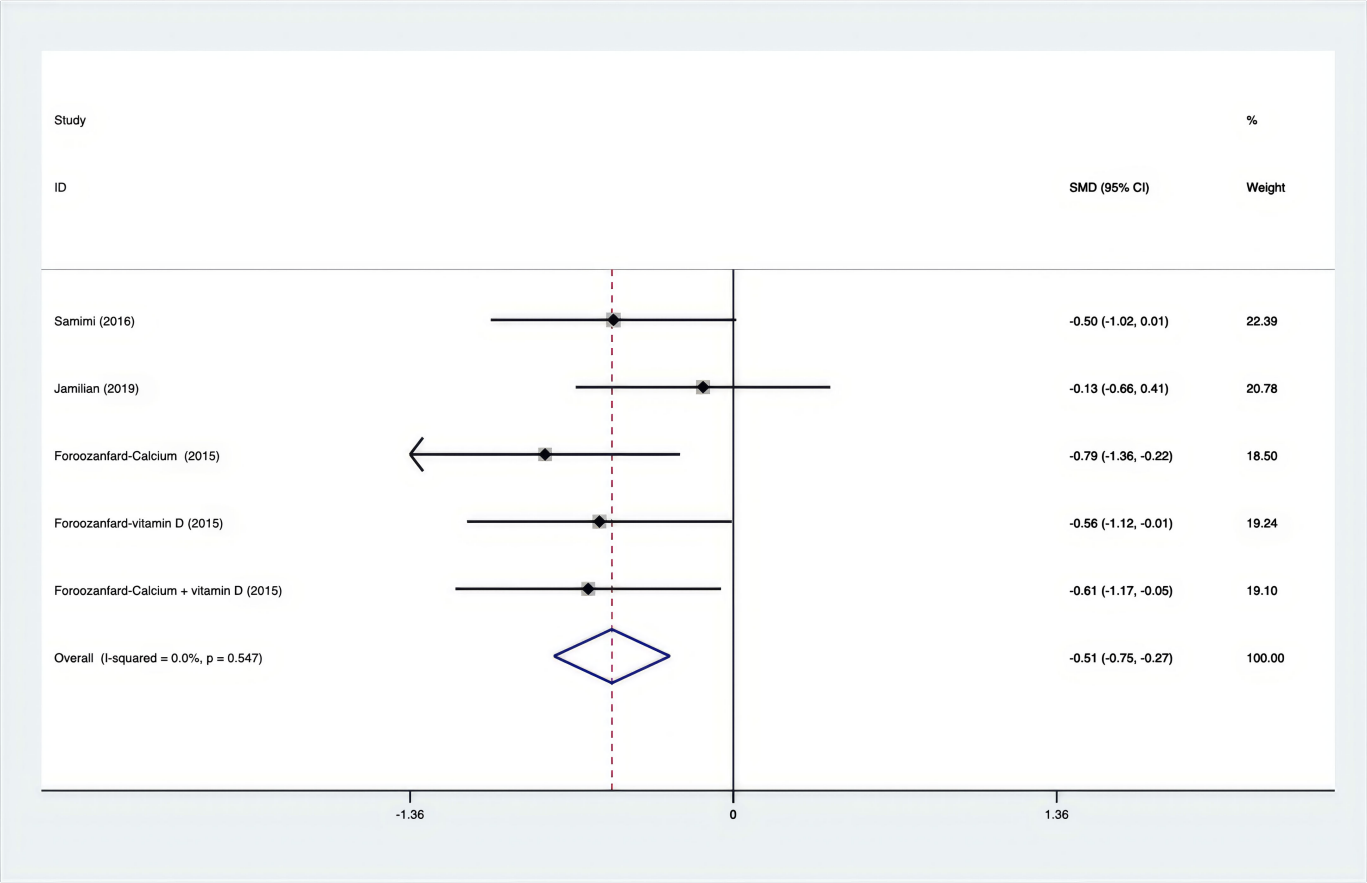
Forest plot of supplements versus placebo on HOMA-B.

**Figure 8:**
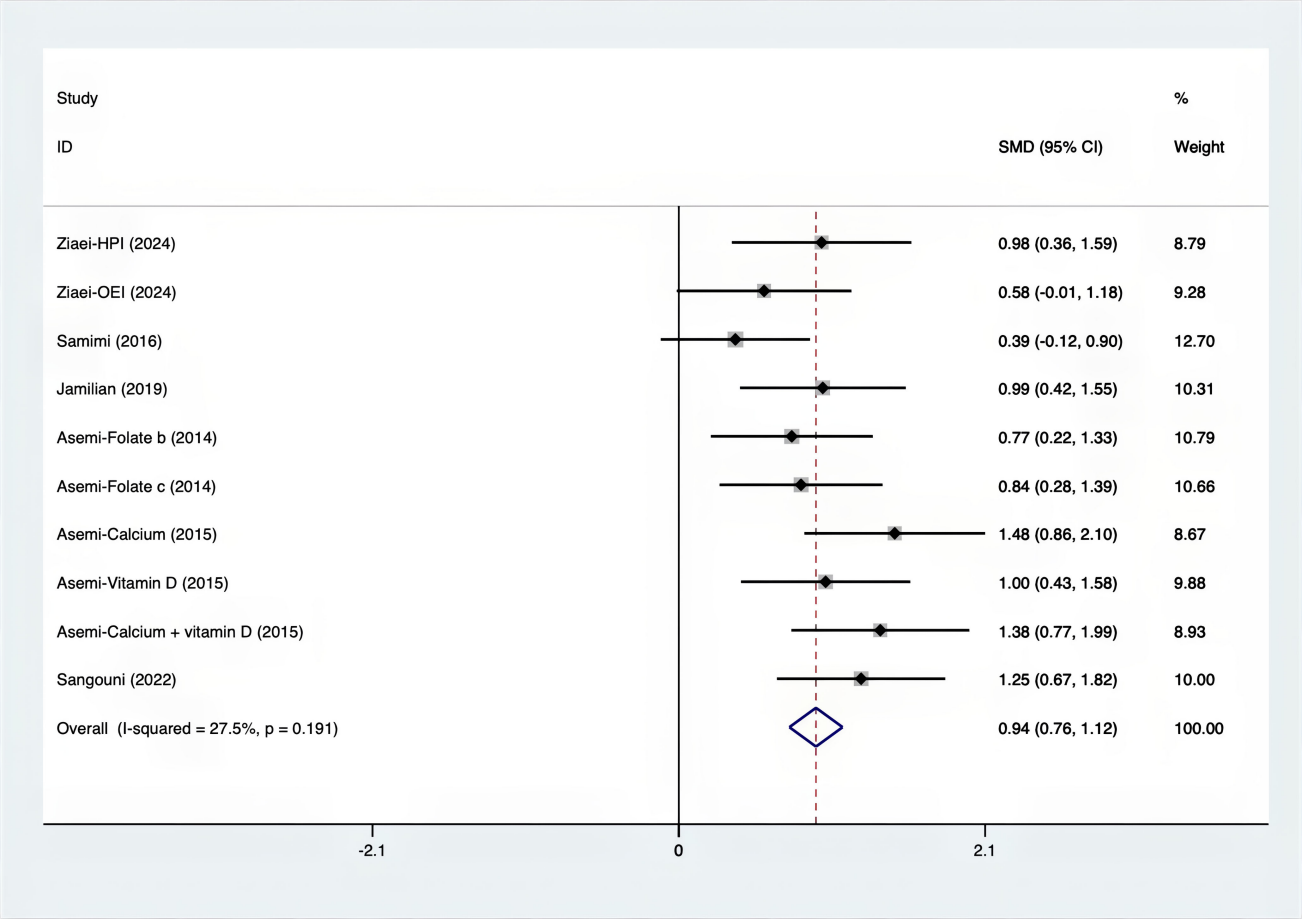
Forest plot of supplements versus placebo on QUICKI.

As shown in **Figures 3**–**8**, both the FPG, insulin, FI, HOMA-IR, HOMA-B, and QUICKI measurements were affected by the difference between the supplements and the placebo. In terms of FPG, insulin, FI, HOMA-IR, HOMA-B, and QUICKI, there was a statistically significant difference between the groups receiving the supplement and the group receiving the placebo. According to the SMD, for FPG it was -0.34 (95% CI, -0.49 to -0.19, p=0.123, I^2^ = 30.8%); for insulin -0.67 (95% CI, -0.83 to -0.52, p=0.208, I^2^ = 24%); for FI -0.26 (95% CI, -0.52 to -0.00, p=0.269, I^2^ = 21.9%); for HOMA-IR -0.59 (95% CI, -0.73 to -0.45, p=0.015, I^2^ = 48.7%); for HOMA-B -0.51 (95% CI, -0.75 to -0.27, p=0.547, I^2^ = 0%); and for QUICKI 0.94 (95% CI, 0.76 to -1.12, p=0.191, I^2^ = 27.5%). Based on these findings, compared with the use of a placebo, the use of supplements was associated with greater improvement in the markers of FPG, insulin, FI, HOMA-IR, and HOMA-B.

#### 3.3.2 Hormonal functions related index

**Figure 9:**
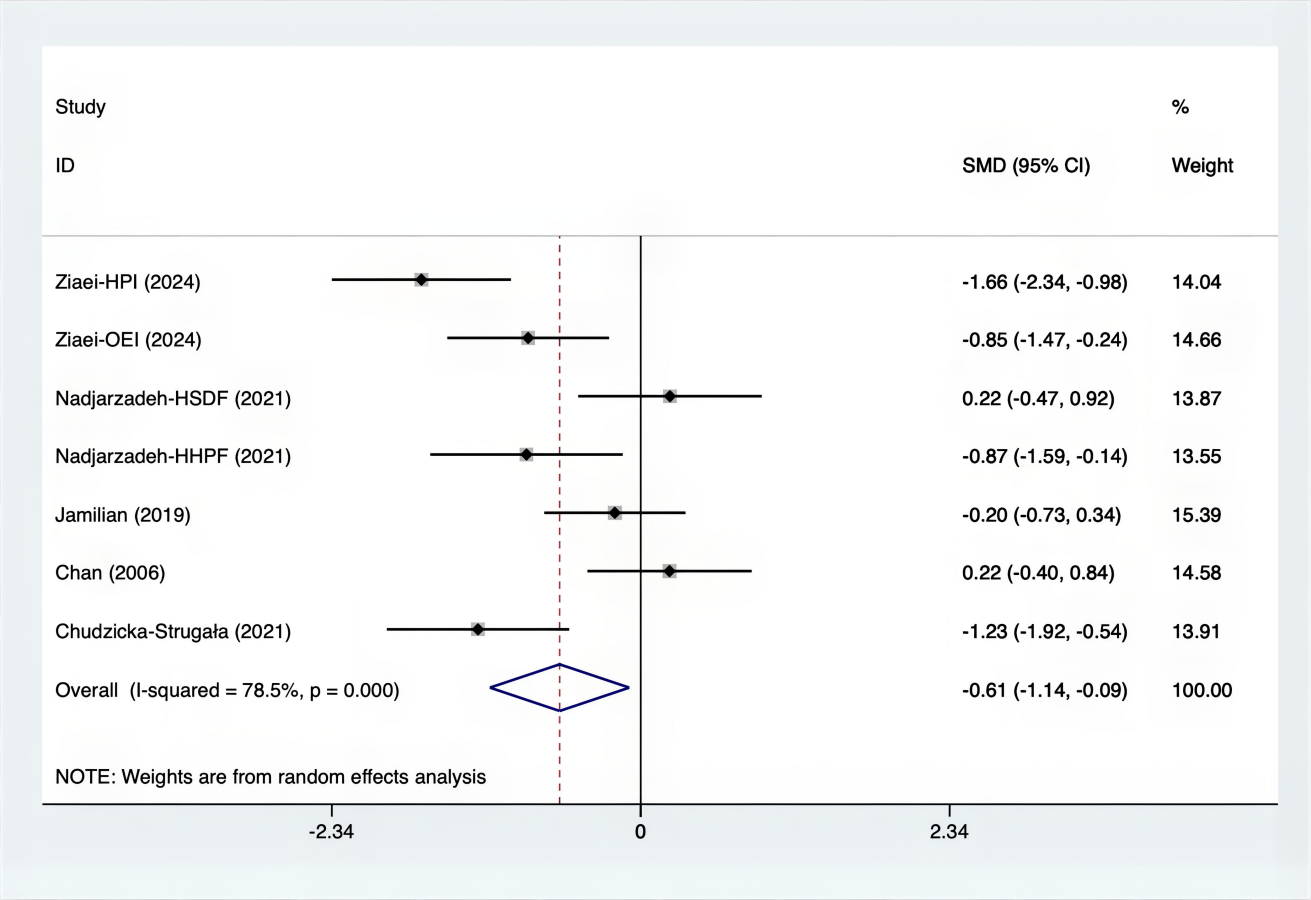
Forest plot of supplements versus placebo on total testosterone.

**Figure 10:**
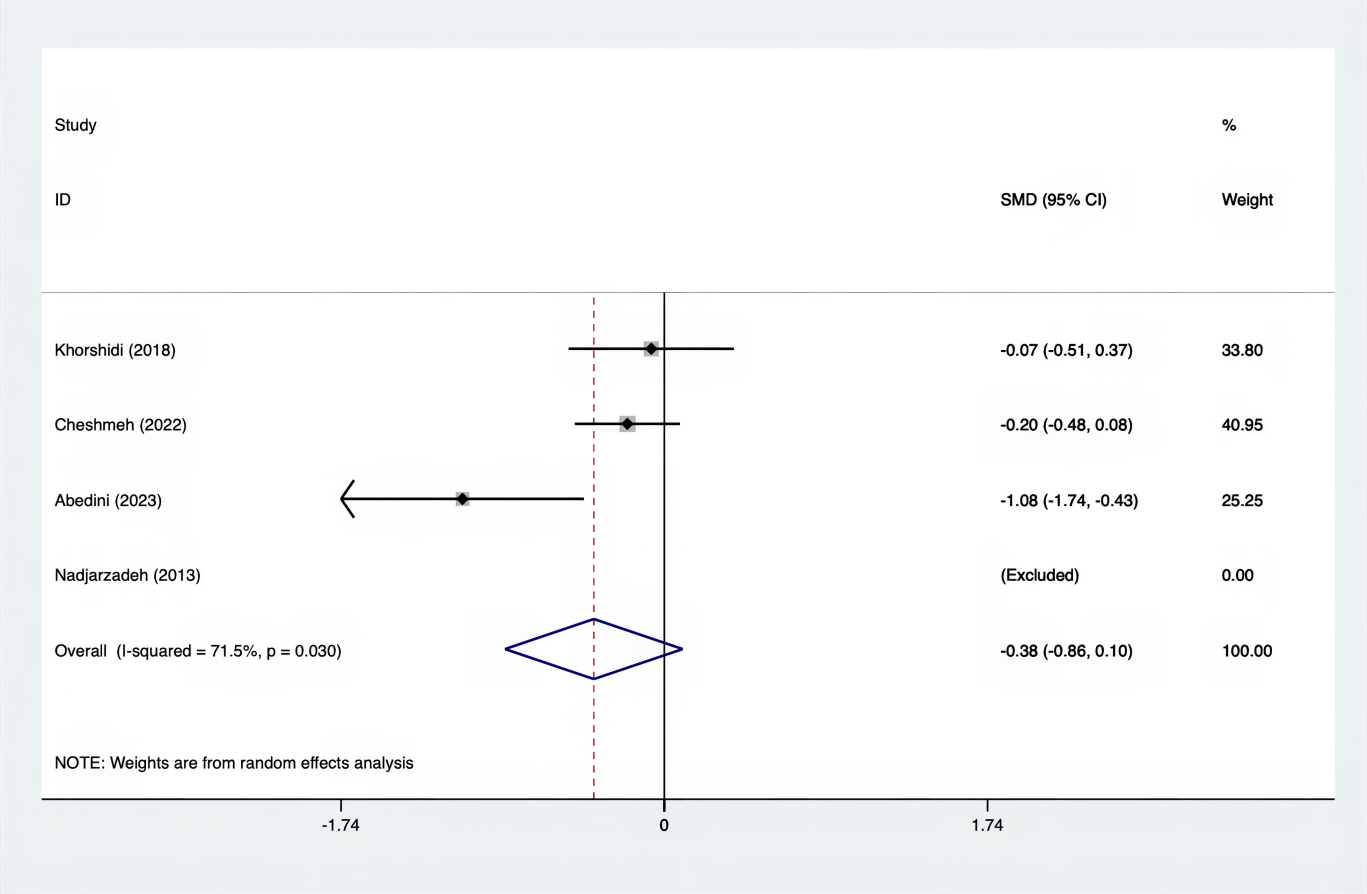
Forest plot of supplements versus placebo on testosterone.

**Figure 11:**
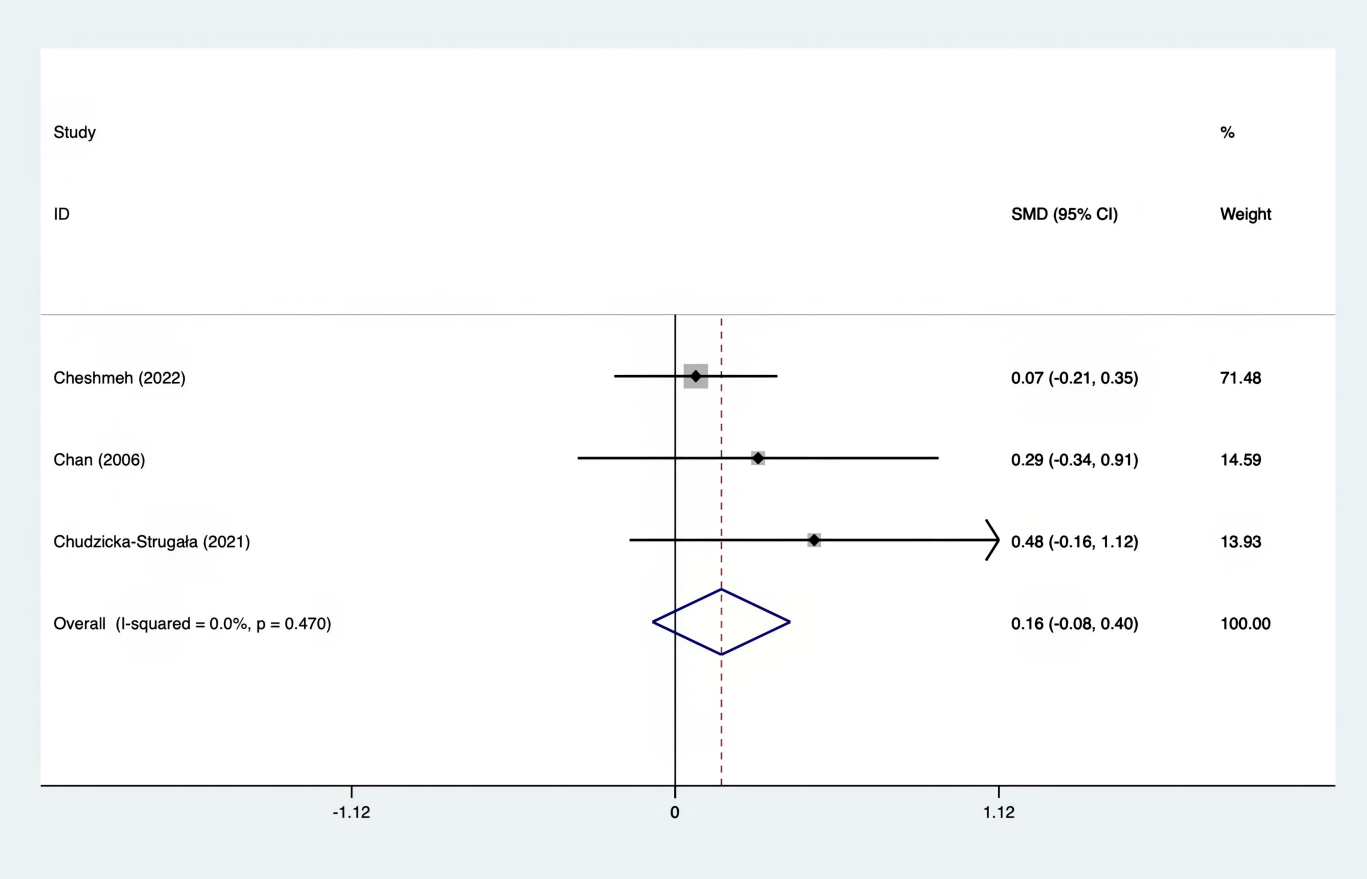
Forest plot of supplements versus placebo on FSH.

**Figure 12:**
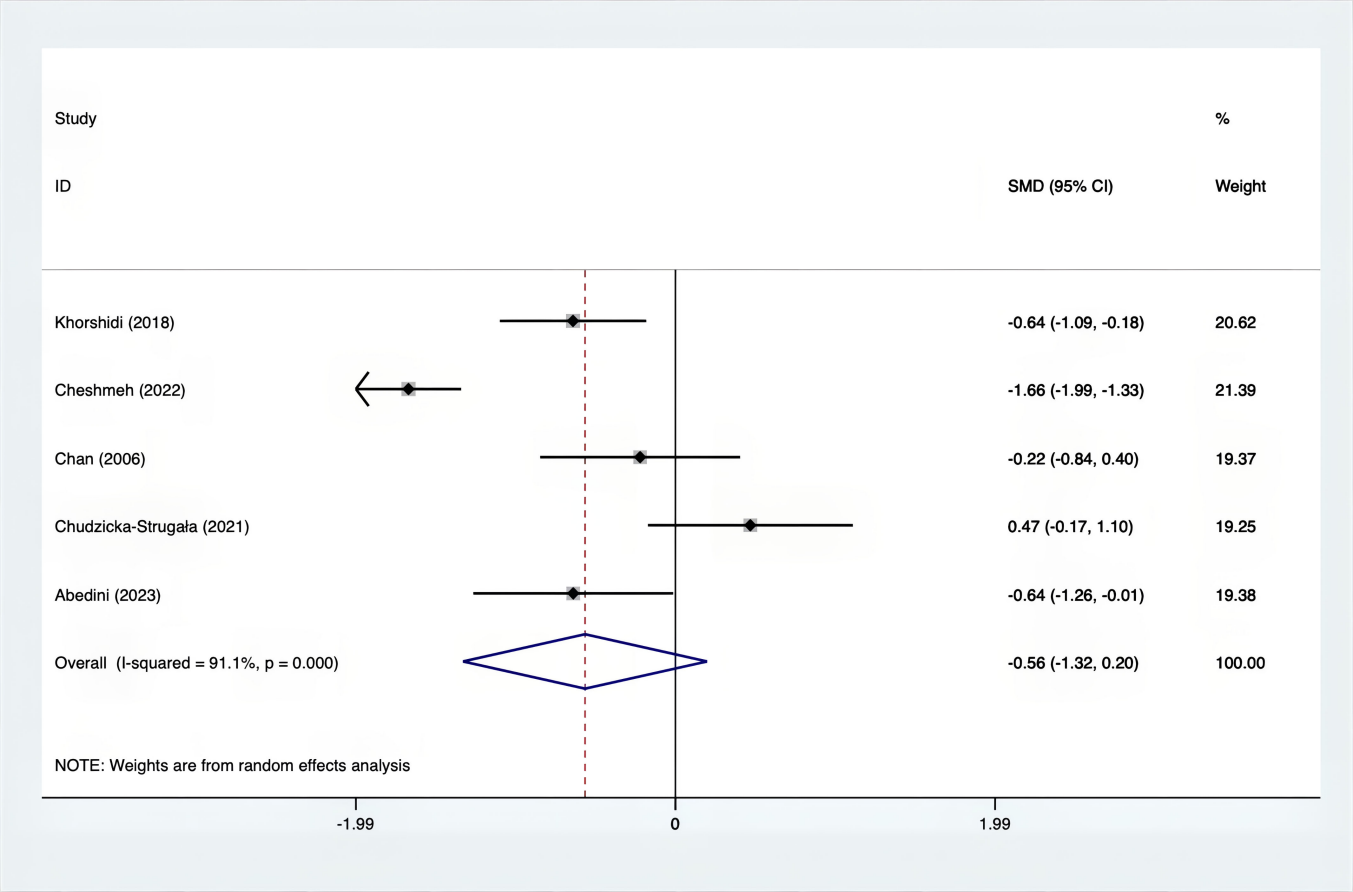
Forest plot of supplements versus placebo on LH.

**Figure 13:**
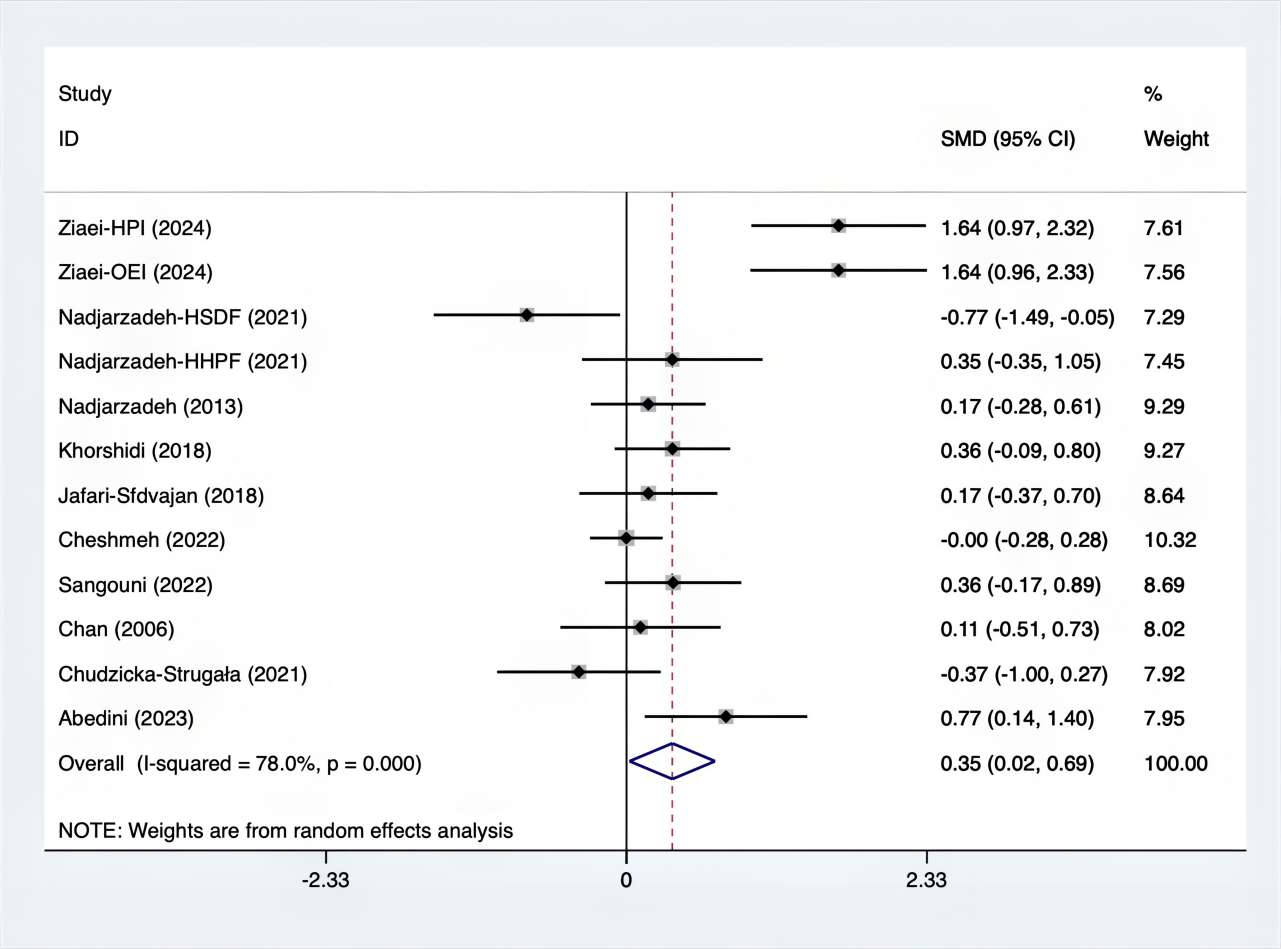
Forest plot of supplements versus placebo on SHBG.

**Figure 14:**
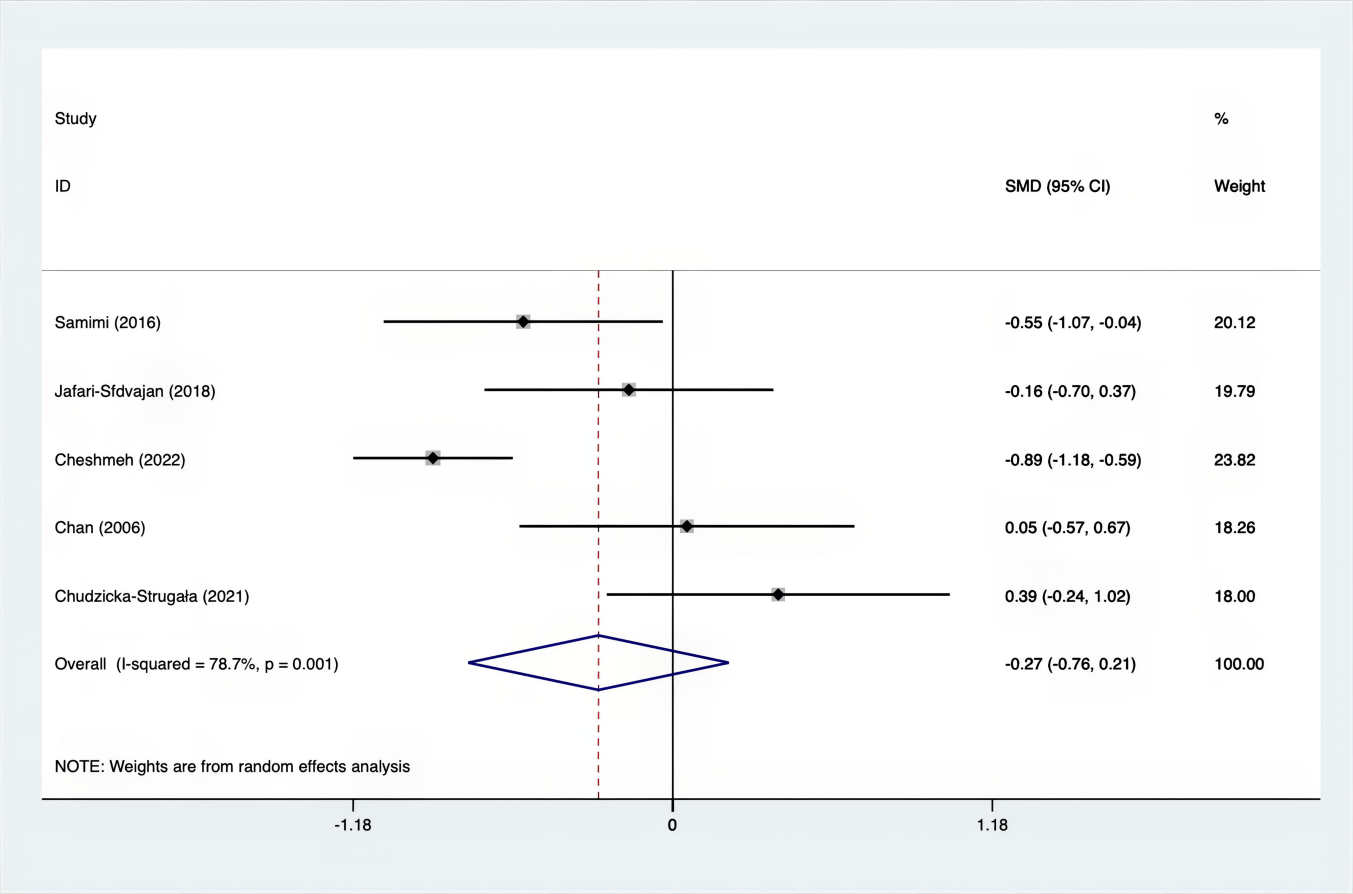
Forest plot of supplements versus placebo on DHEAS.

The SMD values for Total Testosterone, Testosterone, FSH, LH, SHBG, and DHEAS were provided in **Figures 9**–**14**. The effect sizes and statistical significance for the various hormones were as follows: Total Testosterone had an effect size of -0.61 (95% CI, -1.14 to -0.09, p=0.00, I^2^ = 78.5%); Testosterone had an effect size of -0.38 (95% CI, -0.86 to 0.10, p=0.03, I^2^ = 71.5%); FSH had an effect size of 0.16 (95% CI, -0.08 to 0.40, p=0.470, I^2^ = 0%); LH had an effect size of -0.56 (95% CI, -1.32 to 0.20, p=0.000, I^2^ = 91.1%); SHBG had an effect size of 0.35 (95% CI, 0.02 to 0.69, p=0.000, I^2^ = 78%); and DHEAS had an effect size of -0.27 (95% CI, -0.76 to 0.21, p=0.001, I^2^ = 78.7%). Supplements were more effective than placebo in reducing Total Testosterone levels and increasing SHBG levels. A sensitivity analysis was carried out by systematically removing each study one by one (see **Supplementary Material S1**). The analytical results showed the robustness of the findings, remaining consistent throughout the entire process.

#### 3.3.3 Oxidative stress related index

**Figure 15:**
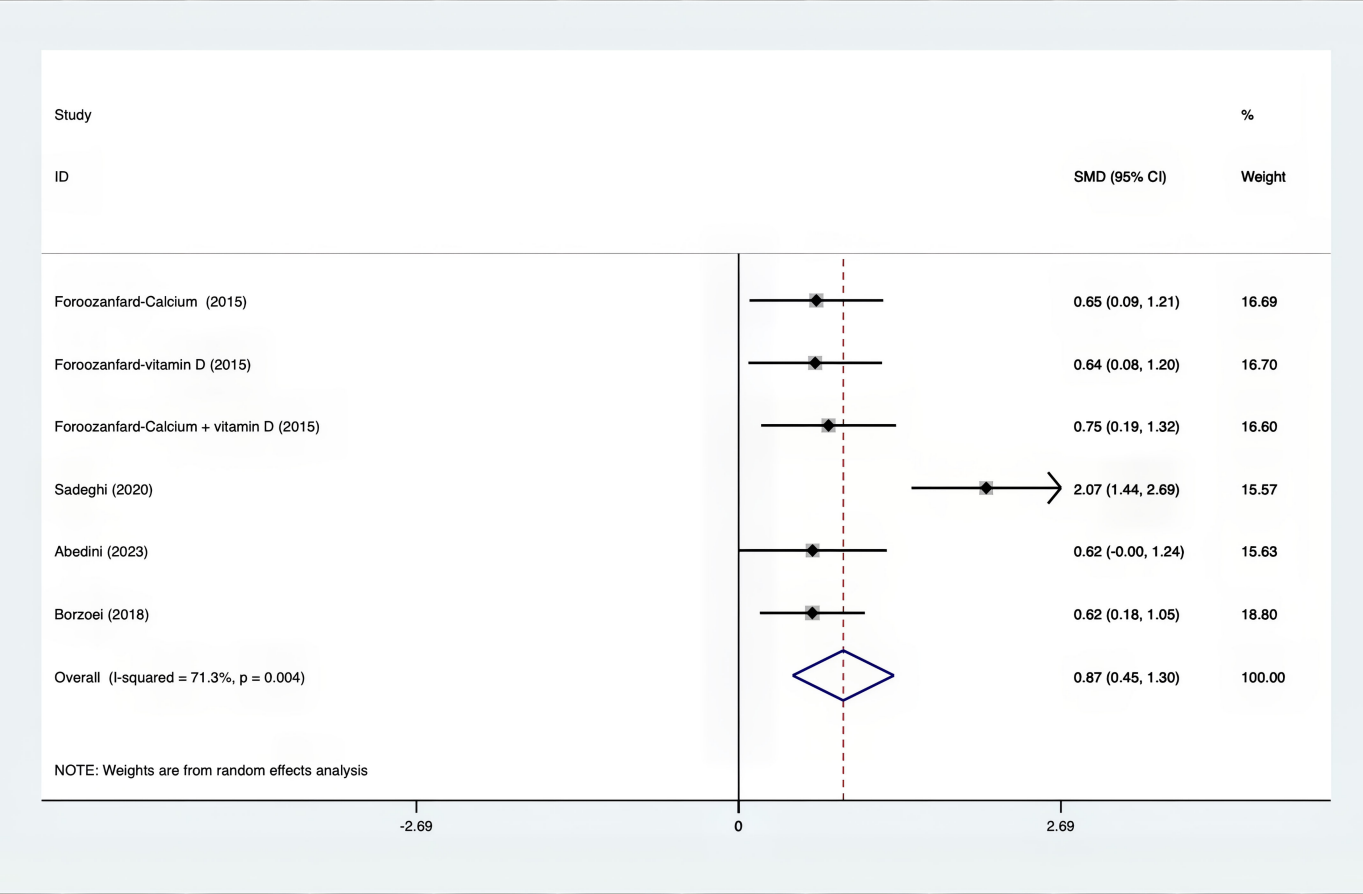
Forest plot of supplements versus placebo on TAC.

**Figure 16:**
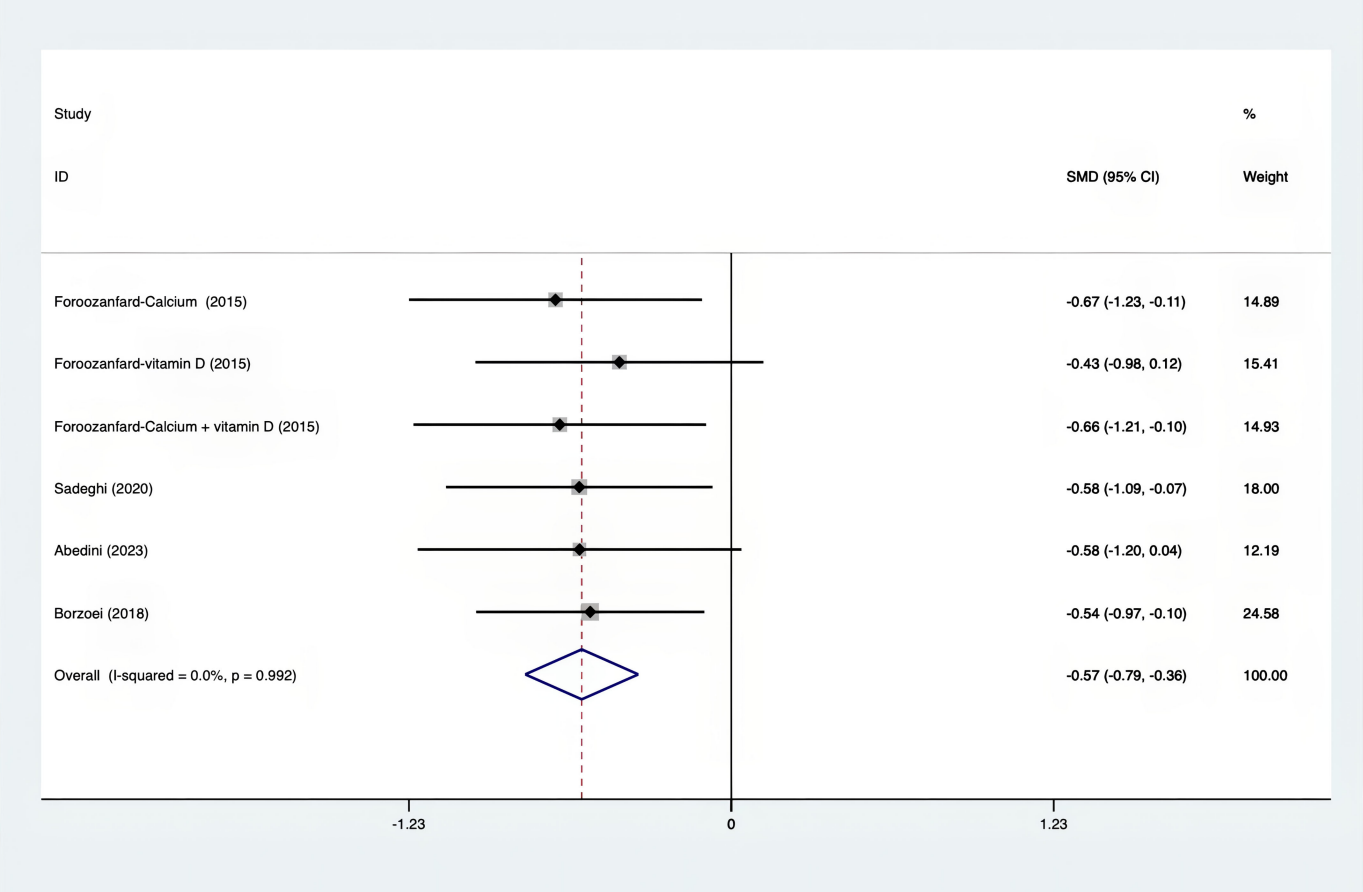
Forest plot of supplements versus placebo on MDA.

The following was a list of the SMD of TAC and MDA, which were presented in **Figures 15**, **16**: MDA was found to have a value of -0.57 (95% CI, -0.79 to -0.36, p=0.992, I^2^ = 0.0%), whereas TAC had a value of 0.87 (95% CI, 0.45 to 1.30, p=0.004, I^2^ = 71.3%). It was proven that supplements were superior to placebo in terms of MDA. A sensitivity analysis was conducted by systematically removing each study one by one in a step-by-step manner (**Supplementary Material S2**). The results of the study were confirmed to be reliable, as they remained consistently unchanged throughout the entire process.

#### 3.3.4 anthropometric outcomes

**Figure 17:**
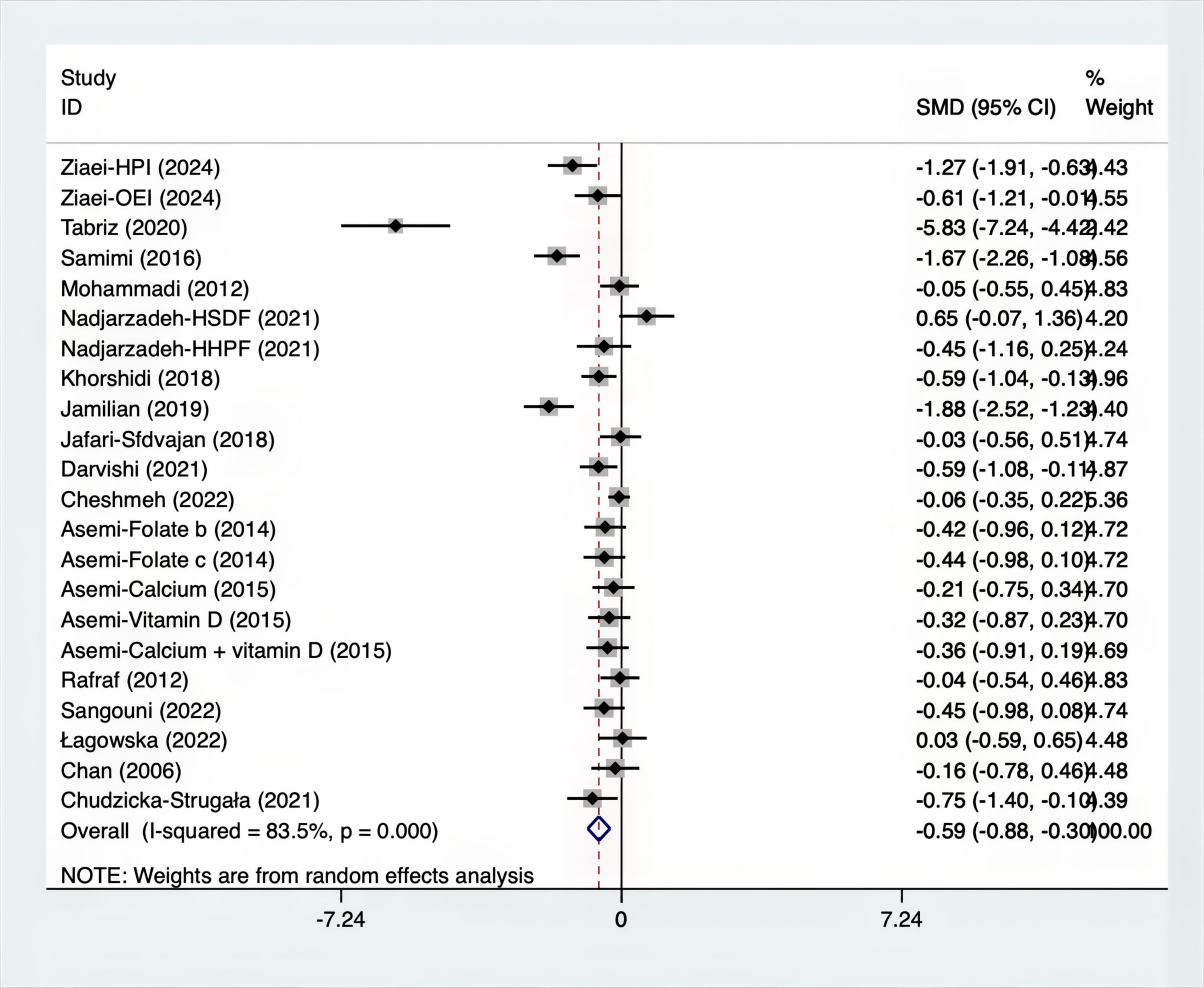
Forest plot of supplements versus placebo on Weight.

**Figure 18:**
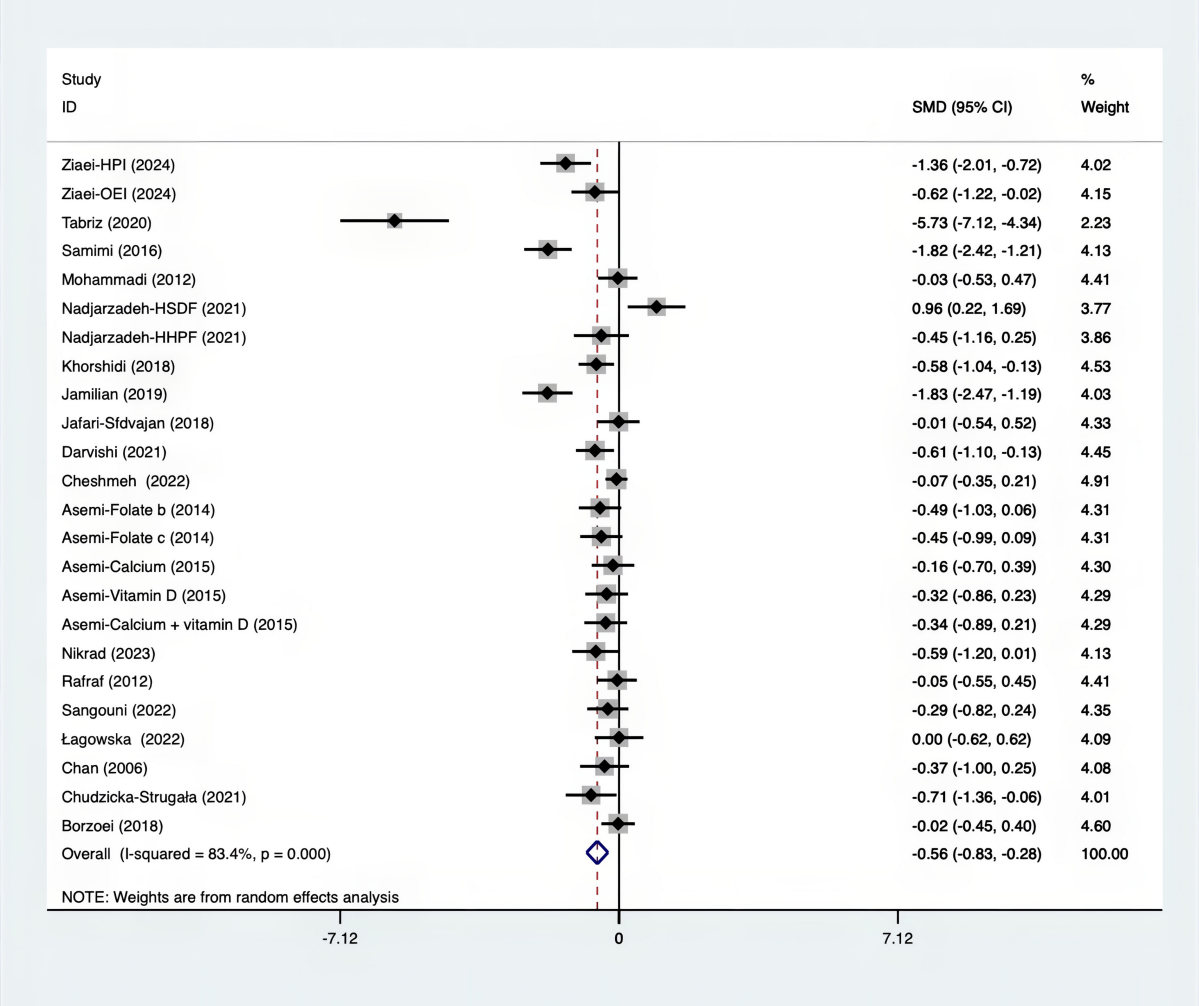
Forest plot of supplements versus placebo on BMI.

**Figure 19:**
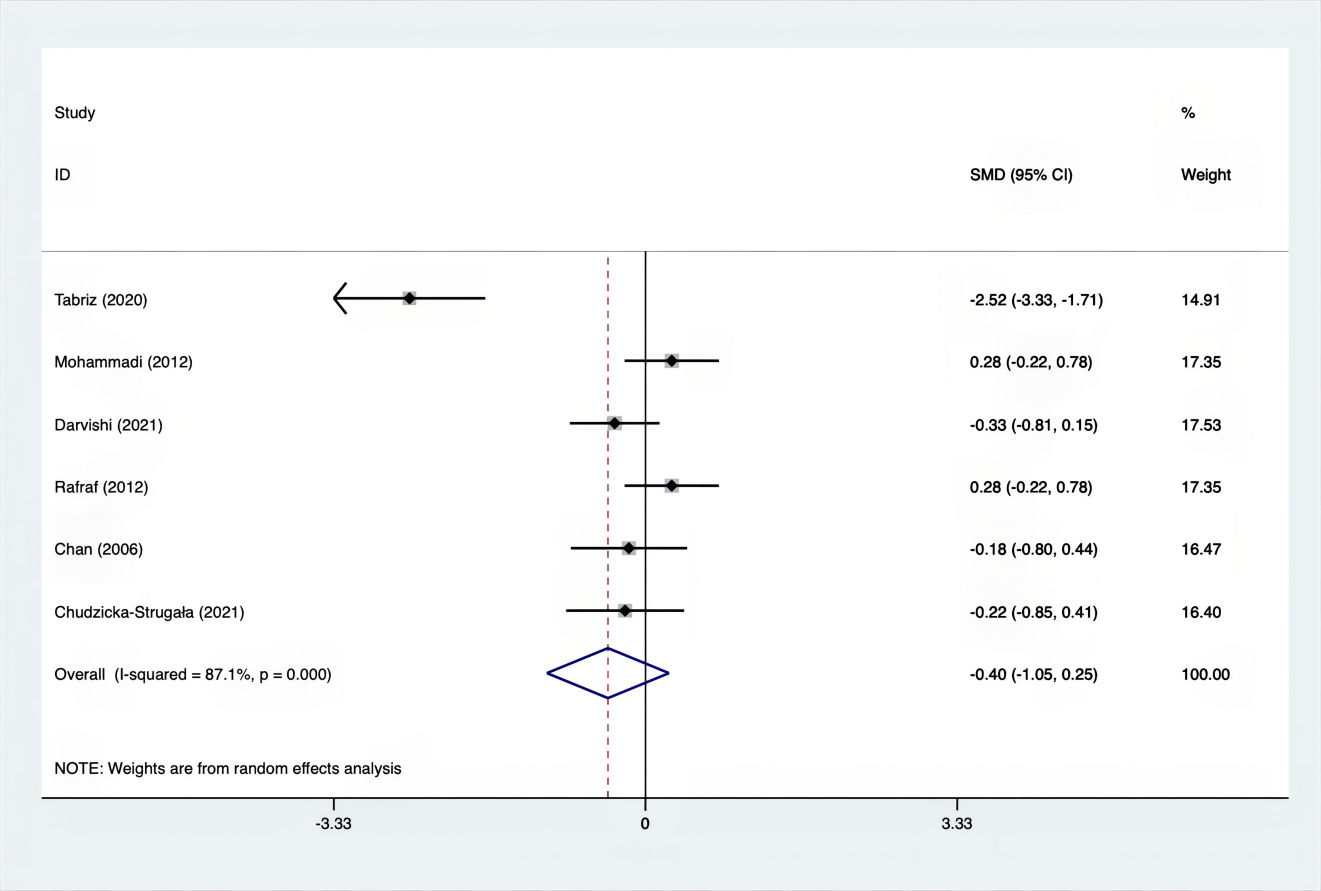
Forest plot of supplements versus placebo on WHR.

**Figure 20:**
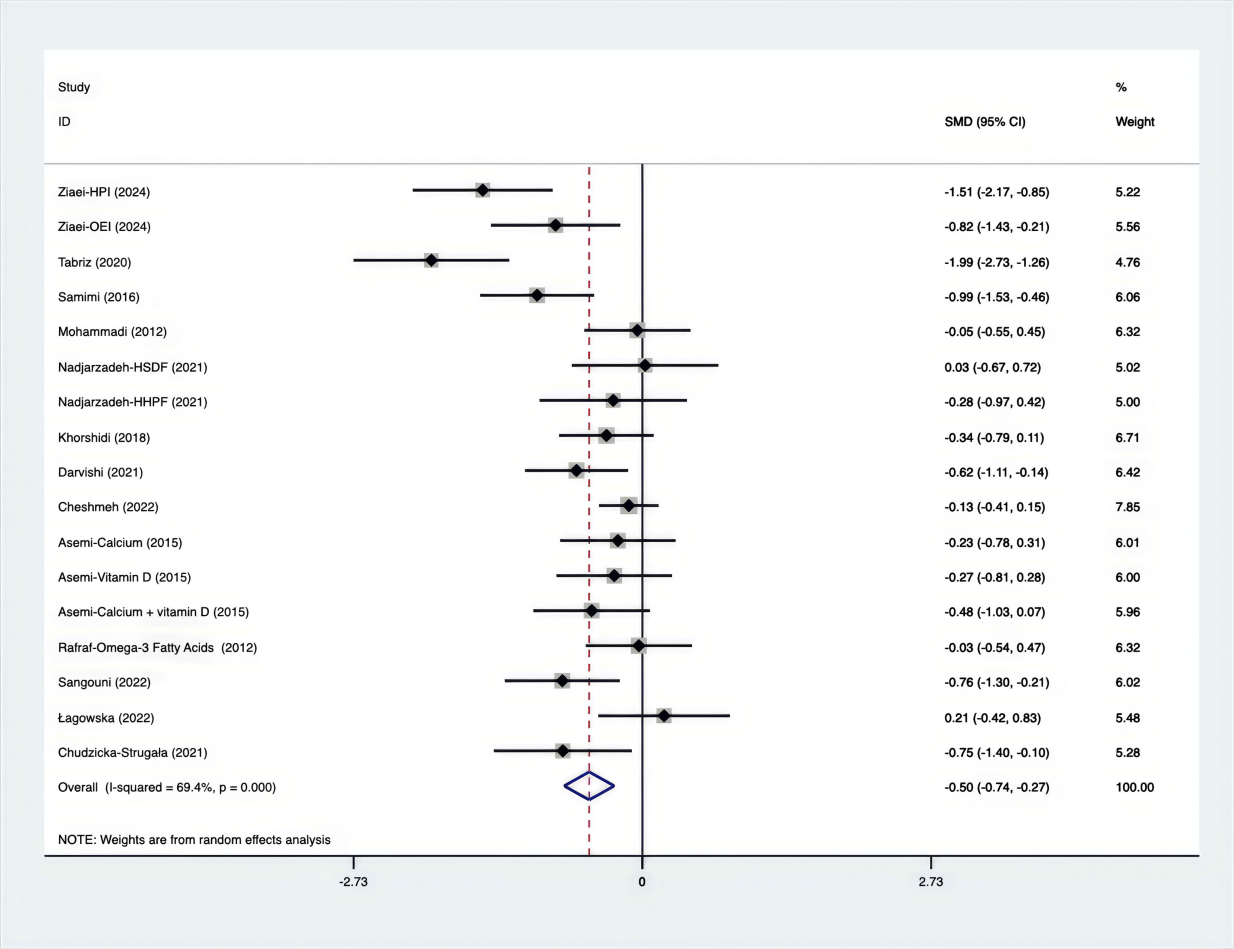
Forest plot of supplements versus placebo on WC.

The SMD for weight, BMI, WHR and WC were as shown in **Figures 17**–**20**. The weight had a negative effect size of -0.59 (95% CI: -0.88 to -0.30, p=0.000, I^2^ = 83.5%). The BMI also had a negative effect size of -0.56 (95% CI: -0.83 to -0.28, p=0.000, I^2^ = 83.4%). The WHR had a relatively smaller negative effect size of -0.40 (95% CI: -1.05 to 0.25, p=0.000, I^2^ = 87.1%). Lastly, the WC had a negative effect size of -0.50 (95% CI: -0.74 to -0.27, p=0.000, I^2^ = 69.4%). Supplements showed outperformed placebo in relation to weight, BMI, WC, and weight. A sensitivity analysis was carried out by systematically excluding each study (**Supplementary Material S3**). The study results were proven to be robust because they remained consistent throughout the entire process.

#### 3.3.5 Ester blood test indicators

**Figure 21:**
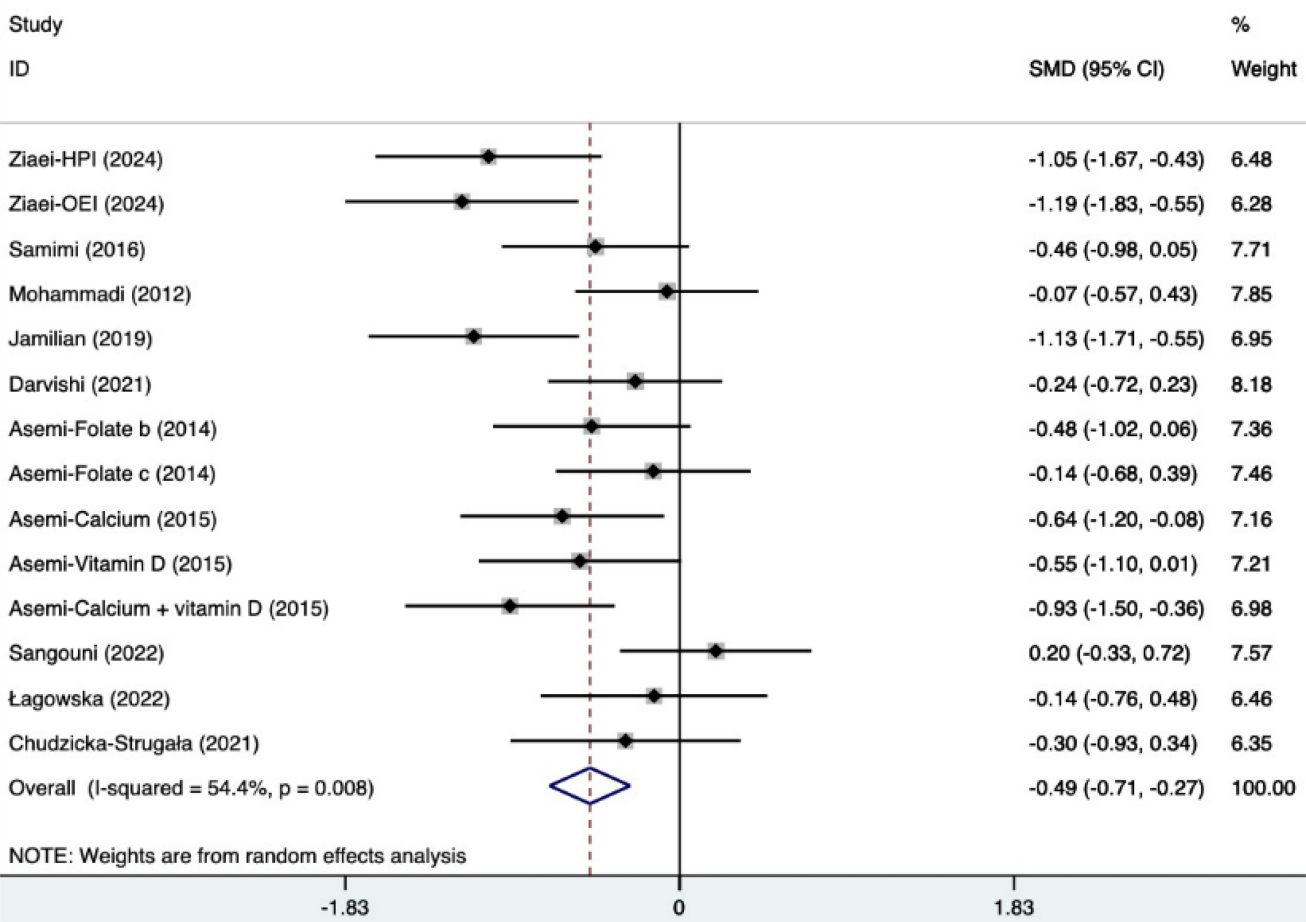
Forest plot of supplements versus placebo on plasma TG.

**Figure 22:**
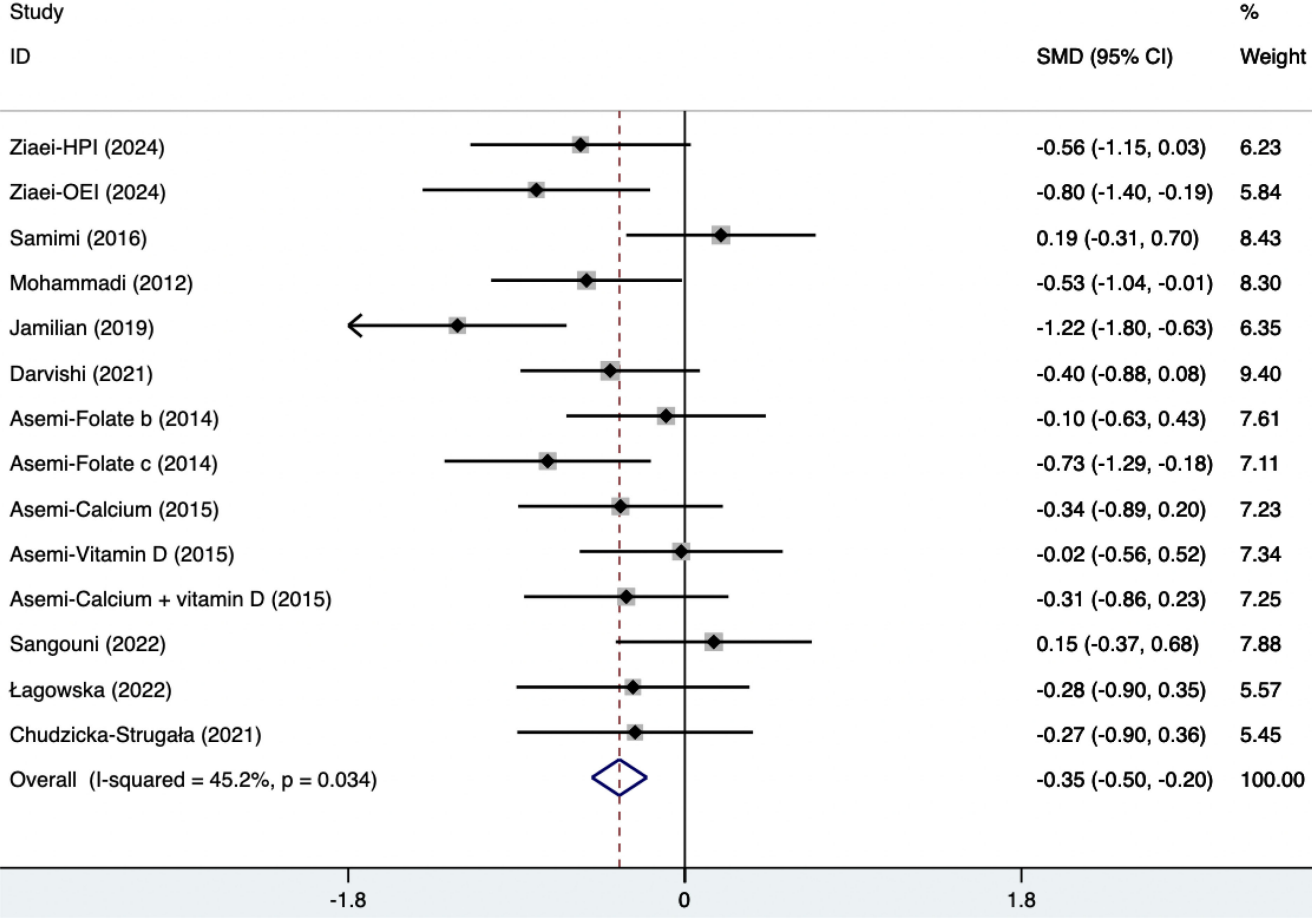
Forest plot of supplements versus placebo on plasma TC.

**Figure 23:**
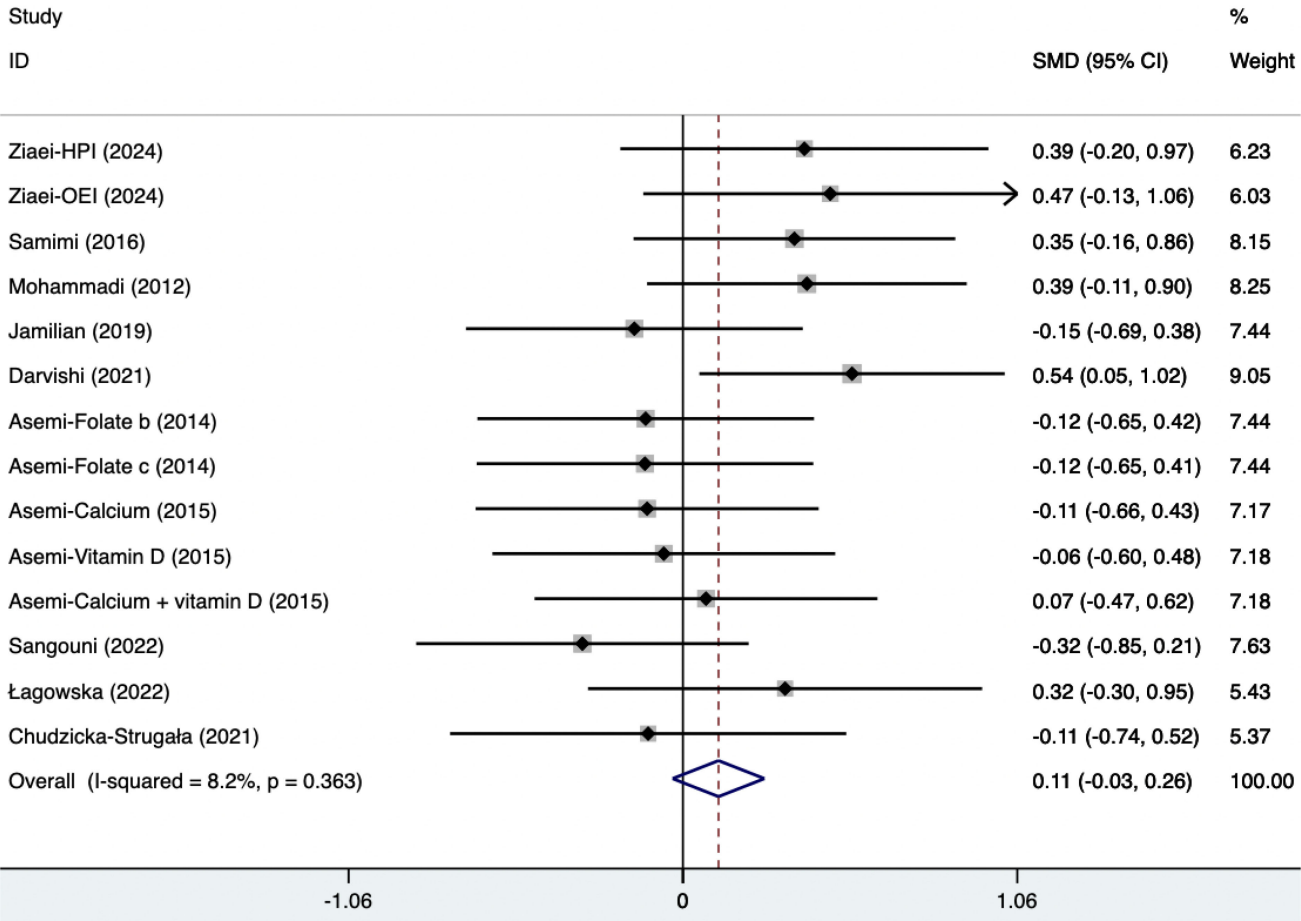
Forest plot of supplements versus placebo on plasma HDL.

The SMD of plasma TG, TC, and HDL were as follows, which were shown in **Figures 21**–**23**: -0.49 (95% CI, -0.71 to -0.27, p=0.008, I^2^ = 54.4%) for plasma TG, -0.35 (95% CI, -0.50 to -0.20, p=0.034, I^2^ = 45.2%) for plasma TC, 0.11 (95% CI, -0.03 to 0.26, p=0.363, I^2^ = 8.2%) for plasma HDL. Supplements were better than placebo in plasma TG, TC, and HDL. A sensitivity analysis was performed by systematically omitting each study one by one (**Supplementary Material S4**). The analysis results showed the durability of the findings, as they remained stable throughout the entire process.

### 3.4 Publication bias

The Egger test was used to assess the presence of publication bias across a range of variables, such as FPG, insulin, FI, HOMA-IR, HOMA-B, QUICKI, DHEAS, Total Testosterone, Testosterone, FSH, LH, SHBG, TAC, MDA, Weight, BMI, WHR, WC, TG, TC, and HDL. The variables Weight (P=0.007), BMI (P=0.006), FPG (P=0.004), QUICKI (P=0.016), TG (P=0.042), DHEAS (P=0.018), and LH (P=0.029) were shown to be more susceptible to publication bias, as indicated by the results presented in **Supplementary Figures 5A–G**. On the other hand, the following outcomes: WC (P=0.062), FI (P=0.574), HOMA-IR (P=0.054), TC (P=0.187), HDL (P=0.746), SHBG (P=0.267), WHR (P=0.056), insulin (P=0.089), HOMA-B (P=0.320), TAC (P=0.338), MDA (P=0.598), Testosterone (P=0.485), Total Testosterone (P=0.402), and FSH (P=0.209) were found to have a lower likelihood of exhibiting publication bias (**Supplementary Figures 6A–N**).

## 4 Discussion

Groundbreaking research has been carried out to investigate the impact of supplements on overweight or obese individuals with PCOS. The inclusion of a greater number of research studies increases confidence in the conclusions, thereby contributing to a more comprehensive data set. Moreover, all the studies included in this paper use the Rotterdam criteria (2003) as the diagnostic criteria for PCOS. This helps to minimize variation in diagnostic criteria and reduce heterogeneity. IR is a crucial factor in the progression and manifestation of PCOS. Severe IR can significantly influence hyperandrogenemia, glucose-metabolism disorders, and ovulation disorders, etc (3). Additionally, when using contraceptives as part of medical treatments for PCOS in obese patients (4), caution should be taken, as hypoglycemic drugs are strongly associated with the occurrence of adverse side effects. Consequently, alternative treatments and adjuvant therapies for PCOS have been explored. In recent years, supplements have shown the potential to improve insulin resistance and alleviate long-term and short-term complications (41).

Compared with placebo, the results of this systematic review and meta-analysis suggest that supplements have a beneficial effect on IR, hyperandrogenism, oxidative stress, BMI, and lipid profiles (13) in overweight or obese individuals with PCOS. These findings are consistent with previous meta-analyses and some RCTs (42–44).

When analyzing supplement types, current evidence suggests that Carnitine, quercetin, Synbiotic, Folate, and Calcium may significantly reduce insulin levels compared to the baseline and placebo. It is also possible that Carnitine, Omega-3, quercetin, Synbiotic, Folate, and Calcium can reduce HOMA-IR relative to the baseline. Calcium might lower HOMA-B relative to the baseline, and Calcium, Omega-3, and Cinnamon could lower MDA relative to the baseline. We speculate that the improvement of insulin resistance by these supplements may be achieved through enhancing glucose and lipid metabolism and reducing oxidative stress (45). For instance, Omega-3 can increase insulin sensitivity by producing and secreting anti-inflammatory adipokines and decreasing inflammation and pro-inflammatory cytokines (46). There was high heterogeneity in Total Testosterone, Testosterone, LH, SHBG, and DHEAS. Further subgroup analysis (**Supplementary Figures 7A–E**) showed that synbiotic and inulin might significantly reduce Total Testosterone relative to the baseline, and concentrated pomegranate juice and inulin could significantly increase SHBG relative to the baseline. Although the forest maps indicated that supplements had no significant effect on Testosterone, LH, and DHEAS, a subgroup analysis (**Supplementary Figures 7B, C, E**) of different supplement types found that concentrated pomegranate juice may significantly reduce Testosterone relative to the baseline, and quercetin, green cardamom, and concentrated pomegranate juice may significantly reduce DHEAS relative to the baseline. We hypothesize that the mechanism by which the above supplements balance sex hormones is complex and may involve the androgen metabolic pathway, oxidative stress, and the balance of intestinal flora. For instance, Anthocyanins and ellagic acid derivatives in concentrated pomegranate juice function as antioxidants (47). Meanwhile, an animal study has shown that its antiandrogenic phenolic compounds may lower testosterone levels by inhibiting DHT receptor complexes and enhancing aromatase activity (48). In addition, prebiotics and probiotics are capable of regulating gut flora, suppressing inflammation, and ameliorating obesity or metabolic disorders, particularly in the bile acid metabolic pathway (49). It should be noted that although these findings appear to offer hope, they need to be interpreted with caution. Mainly because the risk of detection bias is not clear, the sample size is small, and most subgroups only included one study. Consequently, a large number of future studies are required to investigate the effects of supplements on obese PCOS.

The primary goal for managing PCOS is weight loss (44). As per the findings of Balen (50), a 5-10 percent weight loss can effectively restore ovulation and normal menstrual cycles in overweight or obese individuals. This is achieved by reducing blood insulin levels, increasing the levels of SHBG and insulin-like growth factor binding protein, and decreasing blood androgen levels. This article shows evidence that supplements like quercetin, synbiotic, and inulin have been proven effective in promoting weight reduction. However, omega-3, vitamin D, green cardamom, folate, calcium, probiotics, green tea, and thylakoid do not show significant differences from placebo, perhaps because of inconsistent baseline doses. Additionally, given the limited number of participants in these investigations, it may be essential to conduct trials with a larger sample size to get more conclusive results.

PCOS can manifest in a variety of clinical ways, such as infertility, metabolic abnormalities, and psychosocial problems. However, this paper mainly concentrates on endocrine function and metabolic disorders rather than infertility, which is a shortcoming of this study. Moreover, all the participants in this study were overweight or obese, and there were no participants of normal or low weight. Consequently, the results are more relevant to overweight and obese individuals with PCOS. It is crucial to note that endocrine and metabolic dysfunctions also exist in lean or normal-weight individuals with PCOS (5). Thus, it is suggested that future studies should include both obese and lean or normal-weight individuals with PCOS.

### 4.1 Limitations

This evaluation has certain limitations. First of all, the interventions varied greatly in terms of the type, form, dosage, and duration of the supplement, leading to a high degree of variability among the studies. Moreover, there were differences in the initial regulation of nutritional intake, including carbohydrates, proteins, and other components. The observed differences can be attributed to changes in the inclusion criteria, the types of lifestyle interventions used, and the length of these treatments. In view of this, it is important to recognize the potential presence of selection bias, performance bias, and follow-up bias, all of which pose further limitations.

### 4.2 Implications for practice

Recently, there has been an increasing amount of evidence indicating that supplements can be beneficial in enhancing insulin resistance and facilitating weight loss. However, because there are many different types of supplements to choose from and the number of participants in each supplement category is limited, further RCTs are necessary in the future to thoroughly investigate various metabolic markers, such as IR, hyperandrogenemia, and oxidative stress.

## 5 Conclusion

This study represents the first attempt to carry out a systematic review and meta - analysis in order to investigate the effects of supplement therapy on PCOS in overweight or obese individuals. Supplements are more effective than placebo in dealing with insulin resistance, androgenemia, oxidative stress, and lipid status in overweight or obese individuals with PCOS. However, our findings are specifically related to adults and are not applicable to children or the elderly.
