# Supplementary figures and images for: Different supplements improve insulin resistance, hormonal functions, and oxidative stress on overweight and obese women with polycystic ovary syndrome: a systematic review and meta-analysis
Source: Front Endocrinol (Lausanne). 2024 Dec 11;15:1464959. doi: 10.3389/fendo.2024.1464959 (PMC11668966; doi:10.3389/fendo.2024.1464959)

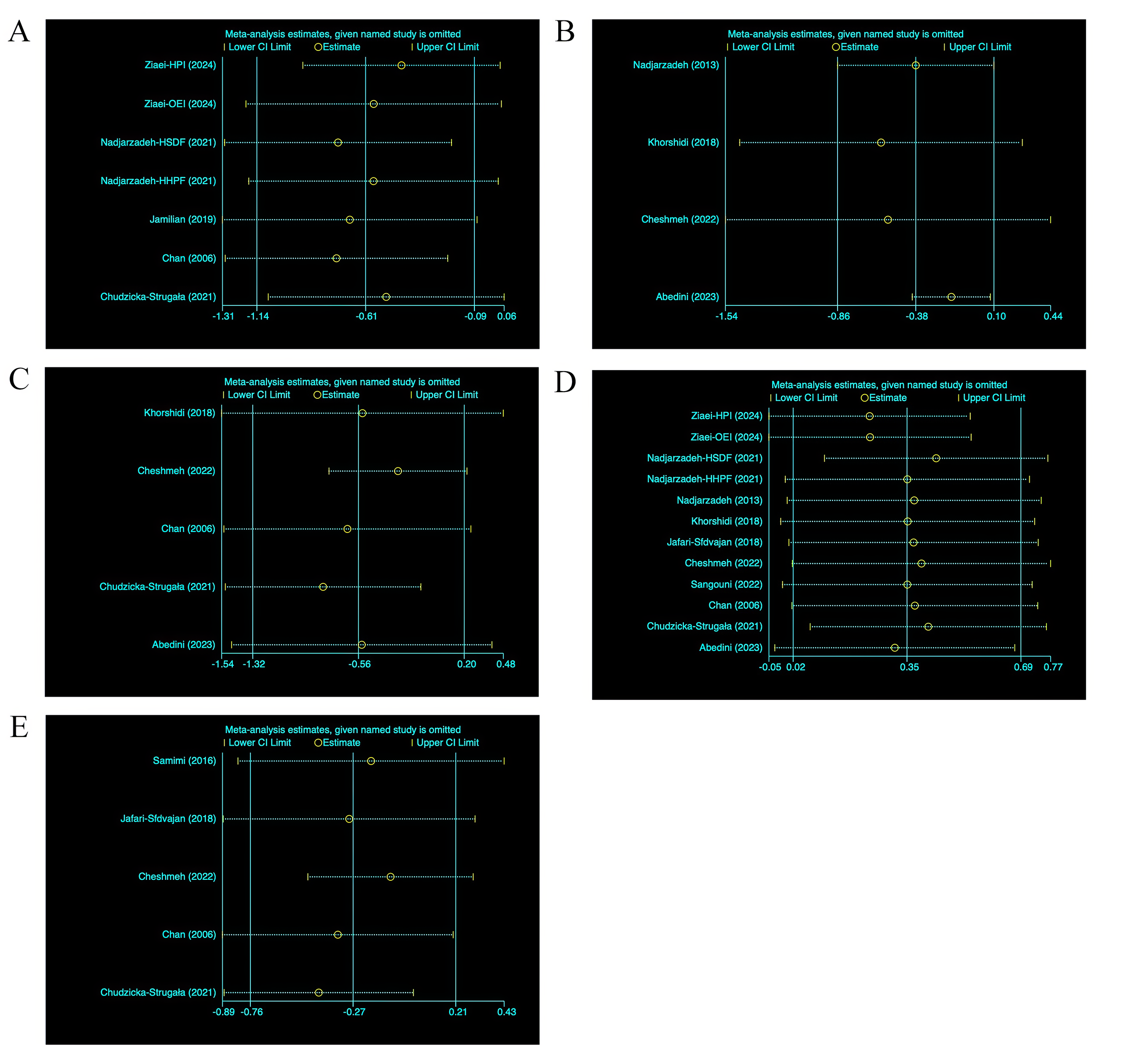

Supplement: Supplementary file 5 [file Image1.tif]

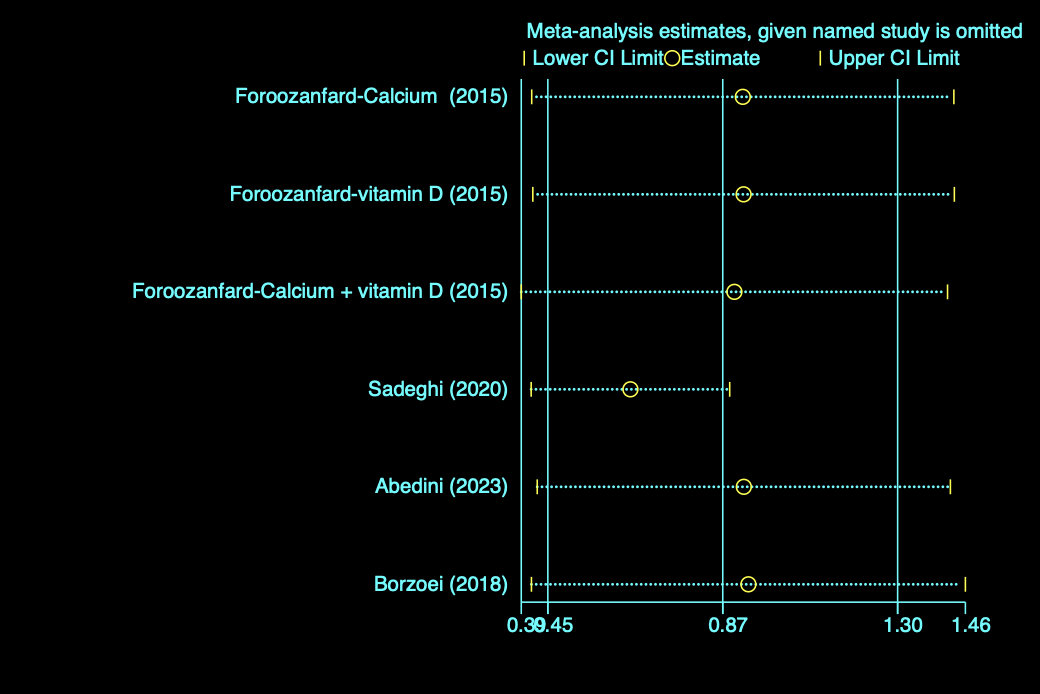

Supplement: Supplementary file 6 [file Image2.png]

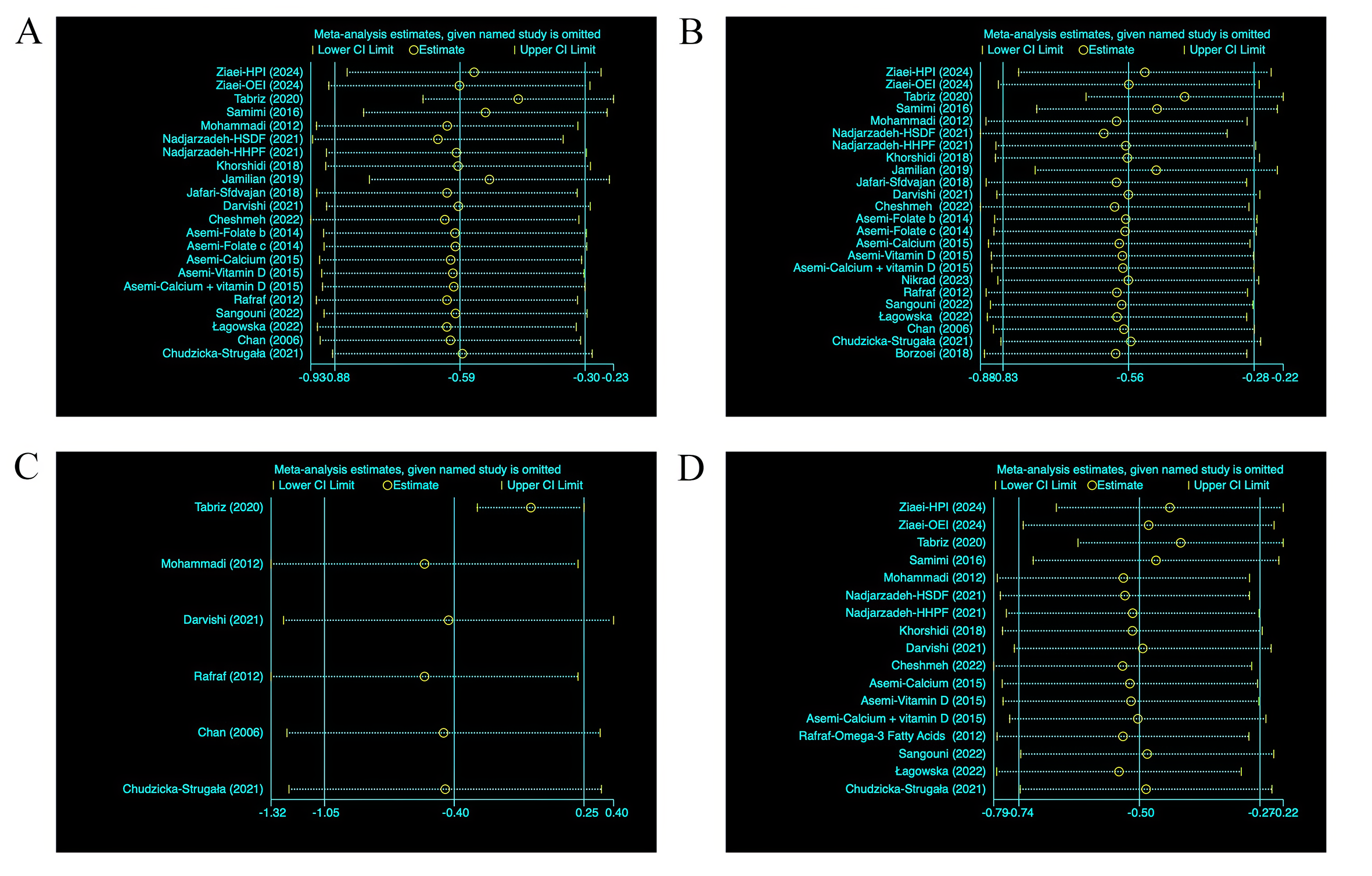

Supplement: Supplementary file 7 [file Image3.tif]

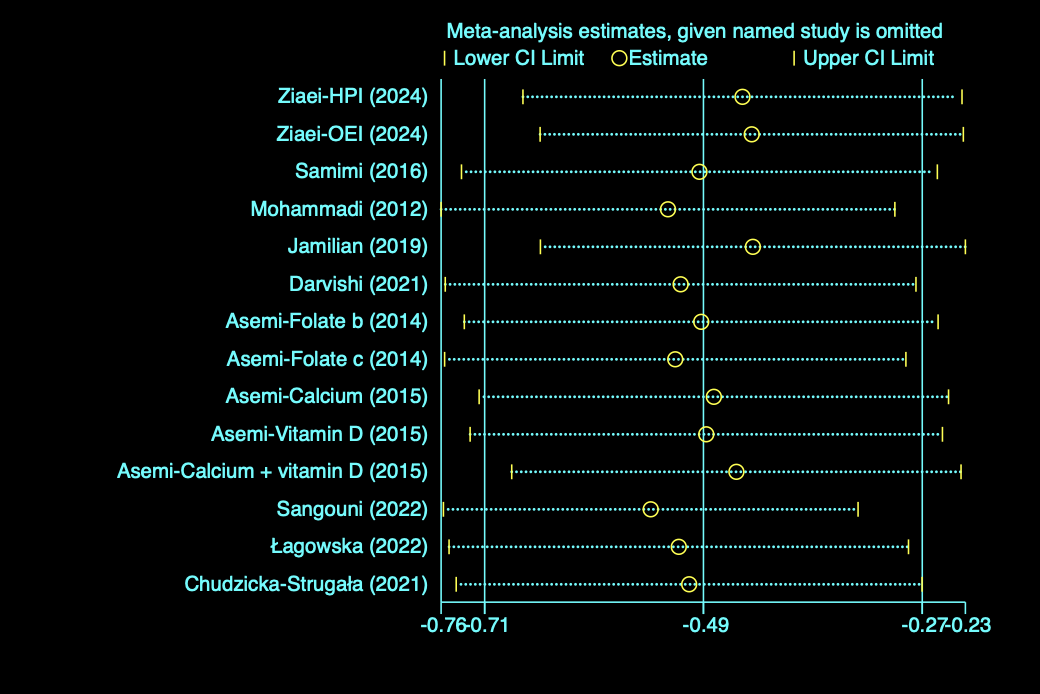

Supplement: Supplementary file 8 [file Image4.png]

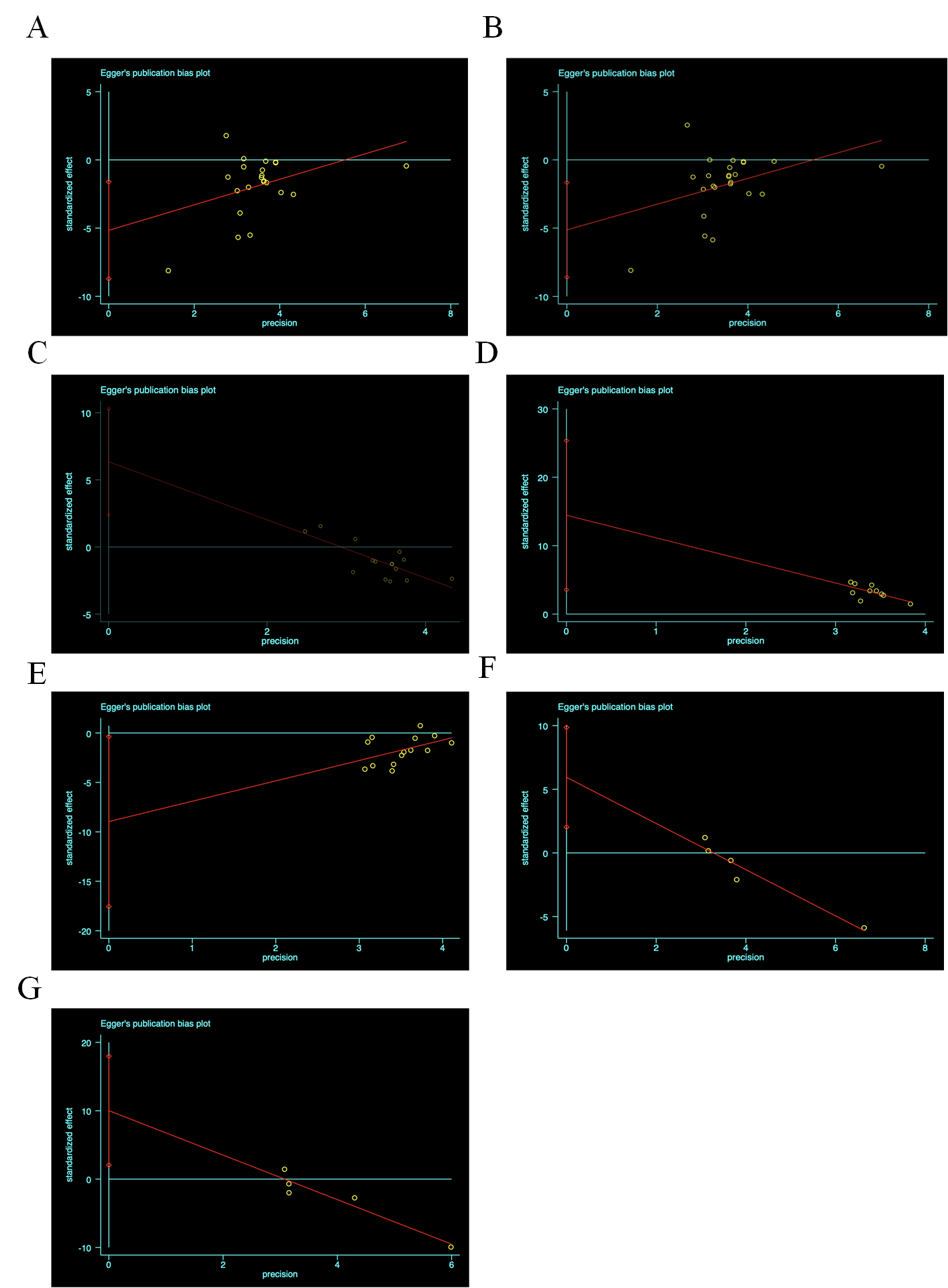

Supplement: Supplementary file 9 [file Image5.png]

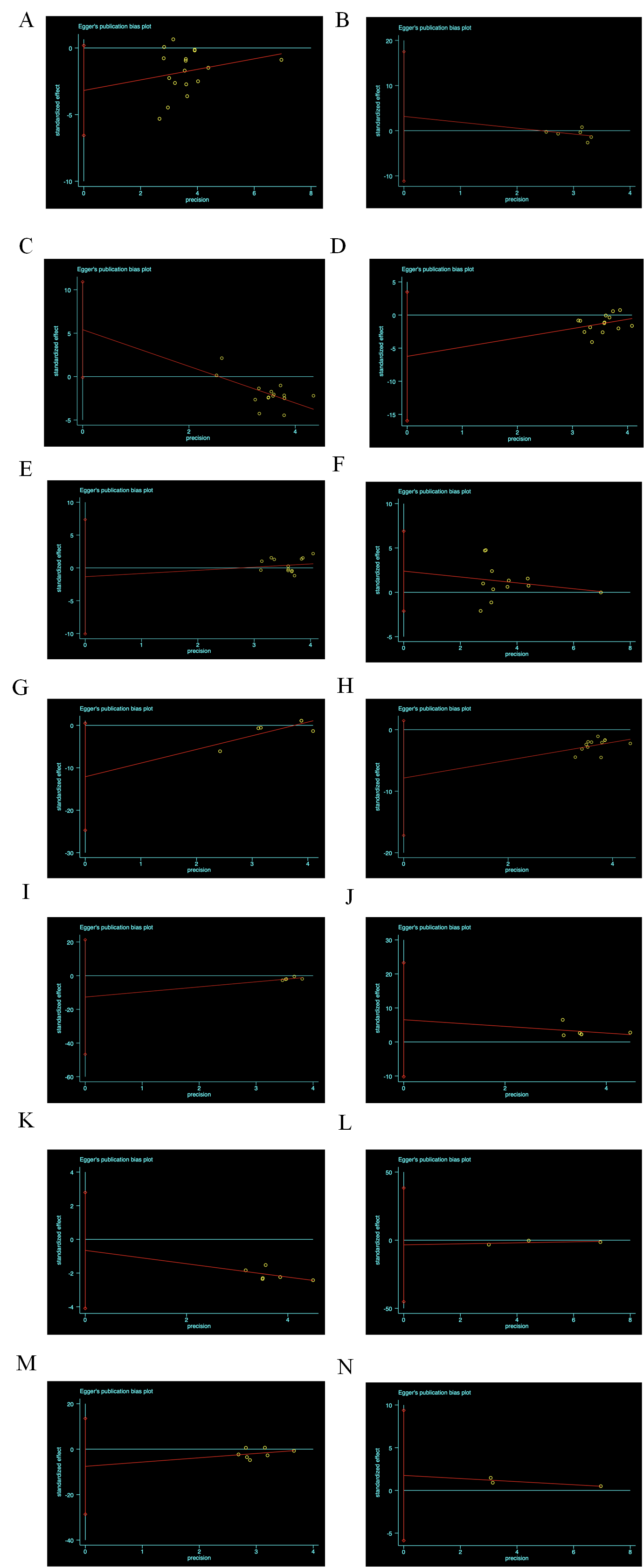

Supplement: Supplementary file 10 [file Image6.png]

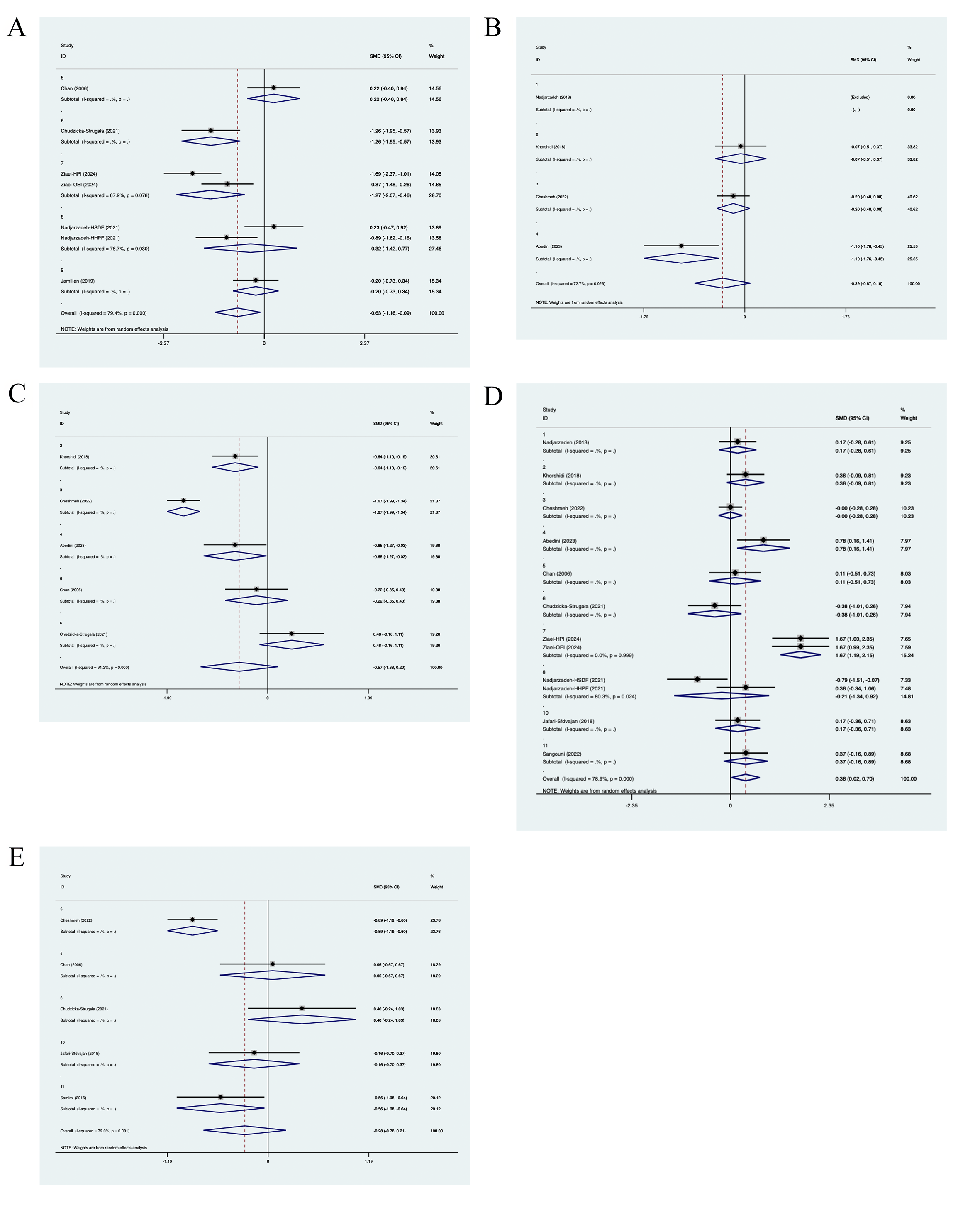

Supplement: Supplementary file 11 [file Image7.tif]
